# Global Health Impact: A Model to Alleviate the Burden and Expand Access to Treatment of Neglected Tropical Diseases

**DOI:** 10.4269/ajtmh.21-0583

**Published:** 2023-02-27

**Authors:** Nicole Hassoun, Leon Cosler

**Affiliations:** ^1^ Department of Philosophy, Binghamton University, Binghamton, New York;; ^2^ Department of Pharmacy Practice, School of Pharmacy and Pharmaceutical Sciences, Binghamton University, Binghamton, New York

## Abstract

Neglected tropical diseases (NTDs) receive relatively little research and development but have a tremendous impact on lifespan and livelihood. Here, we use existing data on the need for drugs, their efficacy, and their treatment percentages to estimate the impacts of various regimens on the global burden of several NTDs: schistosomiasis, onchocerciasis, lymphatic filariasis, and three soil-transmitted helminths (STHs) over time. For an interactive visualization of our models’ results, see https://www.global-health-impact.org/. In 2015, our NTD models estimate that treatment averted 2,778,131.78 disability-adjusted life years (DALYs). Together, treatments targeting STHs together averted 51.05% of the DALYs averted from all NTD treatments, whereas schistosomiasis, lymphatic filariasis, and onchocerciasis medicines averted 40.21%, 7.56%, and 1.18%, respectively. Our models highlight the importance of focusing not just on the burden of these diseases but also on their alleviation in the effort to expand access to treatment.

## INTRODUCTION

To expand access to treatment of neglected tropical diseases (NTDs), it is important to measure the health impact of life-saving drugs on the global burden of these diseases. Worldwide, NTDs, which are defined by the World Health Organization (WHO) as a diverse group of 20 communicable diseases caused by bacteria, viruses, helminths, or protozoa, cause 185,000 deaths a year.^1^ However, this statistic does not take into account the long-term suffering and disability these diseases inflict on over a billion people in poor countries.^2^ Policymakers, pharmaceutical companies, and other stakeholders require information about treatment success in addressing NTD epidemics over time to evaluate performance and allocate resources.

Our NTD models focus in particular on NTDs targeted for elimination—schistosomiasis, onchocerciasis, lymphatic filariasis, roundworm, whipworm, and hookworm—and include many more treatment interventions than most individual disease-based models. NTD interventions are increasingly integrated, targeting more than one disease or group of diseases at once with multiple medications, which suggests that there is a growing need for a modeling framework that allows for the analysis of multiple diseases and drugs.^3^ Most existing models attempt to predict the future course of a single epidemic and treatment efforts’ likely consequences in alleviating a single disease, although studies use a variety of approaches of varying complexity.^4^^–^^6^ Few models assess the impact of multiple pharmaceutical products on a particular disease, opting instead to focus on the efficacy of a single drug on a single disease.^7^^,^^8^

One purported advantage of many existing models is that they are dynamic, but such modeling efforts have several drawbacks. Dynamic models embody a great deal of uncertainty, as they require significant assumptions about the likely developments of epidemics over time (transmission dynamics, etc.). Moreover, many models developed to simulate the transmission and control of NTDs have a restricted geographical scope, frequently being limited to one country or region. These models’ predictions often are not generalizable to other areas.^9^ For example, several models for lymphatic filariasis have only had a modest role in the planning and design of control programs.^10^ Jambulingam et al. produced a model to determine the effectiveness of mass drug administration (MDA) in eradicating lymphatic filariasis in Indian settings, finding that MDA must be continued for longer periods of time in high-transmission areas to be effective.^11^ The model’s predictions could potentially be valid in other nations within the Indian subcontinent, but cannot be used in other areas with differing vectors because of different transmission dynamics.^12^

Although our models emphasize broad epidemiological patterns, they include country-level differences in key parameters such as endemicity to accurately capture burden of disease alleviation within each affected nation and, therefore, globally. Moreover, they have low computational complexity, which is important for our global analysis of five interventions on six NTDs. There is also a need for NTD modeling efforts to incorporate comprehensive disability metrics, such as quality-adjusted life years or disability-adjusted life years (DALYs), to fully capture the disease burden of NTD infections that often have low mortality rates but high disability burdens. Few models use DALYs to estimate the effectiveness of efforts to combat NTDs.^13^^,^^14^ Instead, many models use microfilarial load, average annual number of vector bites received by an adult, or simply disease cases averted.^15^^–^^20^ Utilizing DALY information allows us to create comparable estimates of the interventions’ impacts on disability as well as death over time and across interventions. Moreover, we examine contributions to drug development across the pharmaceutical industry. In short, our models provide a flexible framework for simulating the impact of NTD treatment efforts that can be easily adjusted to reflect new data and standardize results so that impact can be compared across diseases and interventions.

We also provide important information on the pharmaceutical supply chain, given pharmaceutical companies’ differential commitments to provide low-cost or free treatment with a variety of different products. Our data open the door to examining the impact of programs such as Merck’s ivermectin donation program or GlaxoSmithKline’s commitment to donate albendazole to eliminate lymphatic filariasis globally.^21^ Some companies are more generous than others, and our study is the first to try to evaluate the benefits of treatments in estimated DALYs averted.^22^

## MATERIALS AND METHODS

This paper describes a series of models that evaluate the global health consequences of medicines for six NTDs—schistosomiasis, onchocerciasis, lymphatic filariasis, roundworm, whipworm, and hookworm—in 2010, 2013, and 2015.^23^ These diseases were selected because they were targeted for elimination (and based on data availability at the time of model construction). Years were selected to provide a picture of change over time given the available data and for coherence with preexisting global health impact models (https://www.global-health-impact.org).

We use existing data on the need for drugs, their efficacy, and their treatment percentages to estimate the impacts of various treatment regimens on the global burden of our target diseases.^24^ More precisely, we estimate the burden of disease that occurs in the absence of treatment, the impact of drugs on this burden over time, and the contribution of firms’ interventions to alleviating the burden.^24^ The models provide information on the consequences of treatment by company as well as by country, drug, and disease.

The equation below is the impact formula that is used throughout our models to calculate a drug’s impact (measured in DALYs averted) in a single country.

$$I=\frac{D*e*\text{θ}}{1-e*\text{θ}}$$

*D* represents the DALYs observed within the population requiring preventive chemotherapy using data from the Institute for Health Metrics and Evaluation.^25^ *e* represents the efficacy for a specific drug in its respective country. These data were gathered from systematic review of the scientific literature. (See Supplemental Information for a full reference list and this report for the further sources consulted.^24^) θ represents the treatment coverage of a specific drug. It is calculated by dividing the total population treated by the population requiring preventative chemotherapy. These data were gathered using the WHO Preventive Chemotherapy databank.^26^^,^^27^ We use country-level data whenever possible, but in the case of missing data we use regional and, barring that, global fallback data as required (see Discussion and Supplemental Information for data distributions and sensitivity analysis).

To determine which MDA was initiated in each country, we applied two decision trees provided by the WHO’s guidance for preventive chemotherapy in human helminthiasis.^27^ The decision trees specify treatments for each possible epidemiological combination of the diseases our NTD models analyze. The decision trees are illustrated in Supplemental Figures 4 and 5. T1, T2, and T3 refer to a unique targeted treatment (T1 = albendazole + praziquantel or mebendazole + praziquantel, T2 = praziquantel, and T3 = albendazole or mebendazole). MDA1, MDA2, and MDA3 refer to a unique MDA (MDA1 = ivermectin + albendazole, MDA2 = diethylcarbamazine +albendazole, and MDA3 = ivermectin).

The information on specific drugs used to address different NTDs is contained in Supplemental Table 1.^4^ Information on the dosage for each respective anthelmintic drug along with its frequency of intervention is found in Supplemental Table 2.^4^ Information regarding a regimen’s targeted disease and its frequency of implementation is found in Supplemental Table 3.^4^

We gather data on endemicity from the WHO’s Preventive Chemotherapy and Transmission Control databank and assume that having a population requiring treatment of a disease in a given country makes the disease endemic in that country when endemicity is not listed explicitly.^3^ The WHO’s weekly epidemiological record provides a framework to determine soil-transmitted helminths’ (STHs’) level of endemicity for an individual country.^5^ According to the WHO, STHs are highly endemic in a country if the proportion of the population requiring preventive chemotherapy is greater than or equal to two-thirds of preschool- and school-aged children. STHs are moderately endemic if the proportion of the population requiring preventive chemotherapy is between one-third and two-thirds of preschool- and school-aged children. Finally, STHs have low endemicity if the proportion of the population requiring preventive chemotherapy is less than one-third of preschool- and school-aged children. We use information taken from the WHO’s Preventive Chemotherapy and Transmission Control databank to estimate endemicity using these definitions. We sum the population requiring preventive chemotherapy for STHs for preschool- and school-aged children and divide this by the total population of preschool- and school-aged children from the World Bank database.^6^ Supplemental Table 4 describes the recommended treatment strategies based on STH prevalence among school-aged children.^1^

## RESULTS

### The effects of interventions on the burden of disease alleviated.

**Figure 1.**
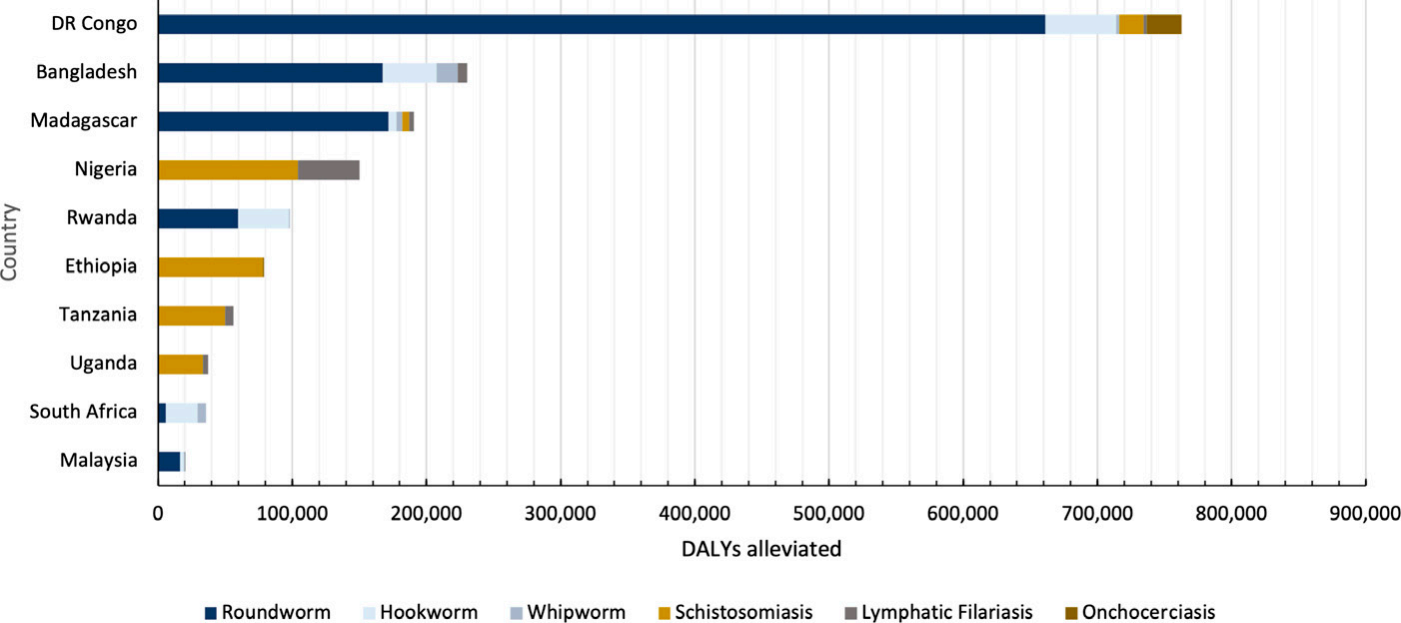
Total disability-adjusted life years (DALYs) alleviated by country. Data show the total DALYs alleviated for all neglected tropical diseases in countries with high impact scores from distinct regions throughout 2015.

Our NTD models estimate the global distribution of DALYs alleviated across countries. Key medicines are having the most impact in Africa and Southeast Asia; the need for STHs and schistosomiasis is highly concentrated in these regions. The marked change in albendazole impact from 2013 to 2015 comes from roundworm intervention in Cameroon—the combination of high efficacy and treatment coverage in 2013 increased impact substantially, but roundworm was not considered endemic to Cameroon in 2015 and so an impact score was not calculated.^27^ Figure 1 shows that drugs for our target diseases are having the greatest impact in the Democratic Republic of Congo. There is a considerable amount of roundworm infection in the Democratic Republic of Congo receiving highly effective treatment. Globally, there are areas with great need but correspondingly little impact. The most glaring example of this failure can be found in South America: the ratio of impact to need in this region is 34.71%. In other words, in 2015, out of 240,625.51 DALYs we estimate would have accrued absent treatment, approximately 83,532.47 DALYs were averted in South America using NTD interventions, leaving 157,093.04 DALYs accrued in South America. Additionally, the models highlight substantial regional disparities in treatment coverage, efficacy, and need. Treatment coverage for schistosomiasis in 2015 is considerably higher in the western Pacific region than in the African region, for instance, even though the majority of schistosomiasis DALYs are located in Africa.

**Figure 2.**
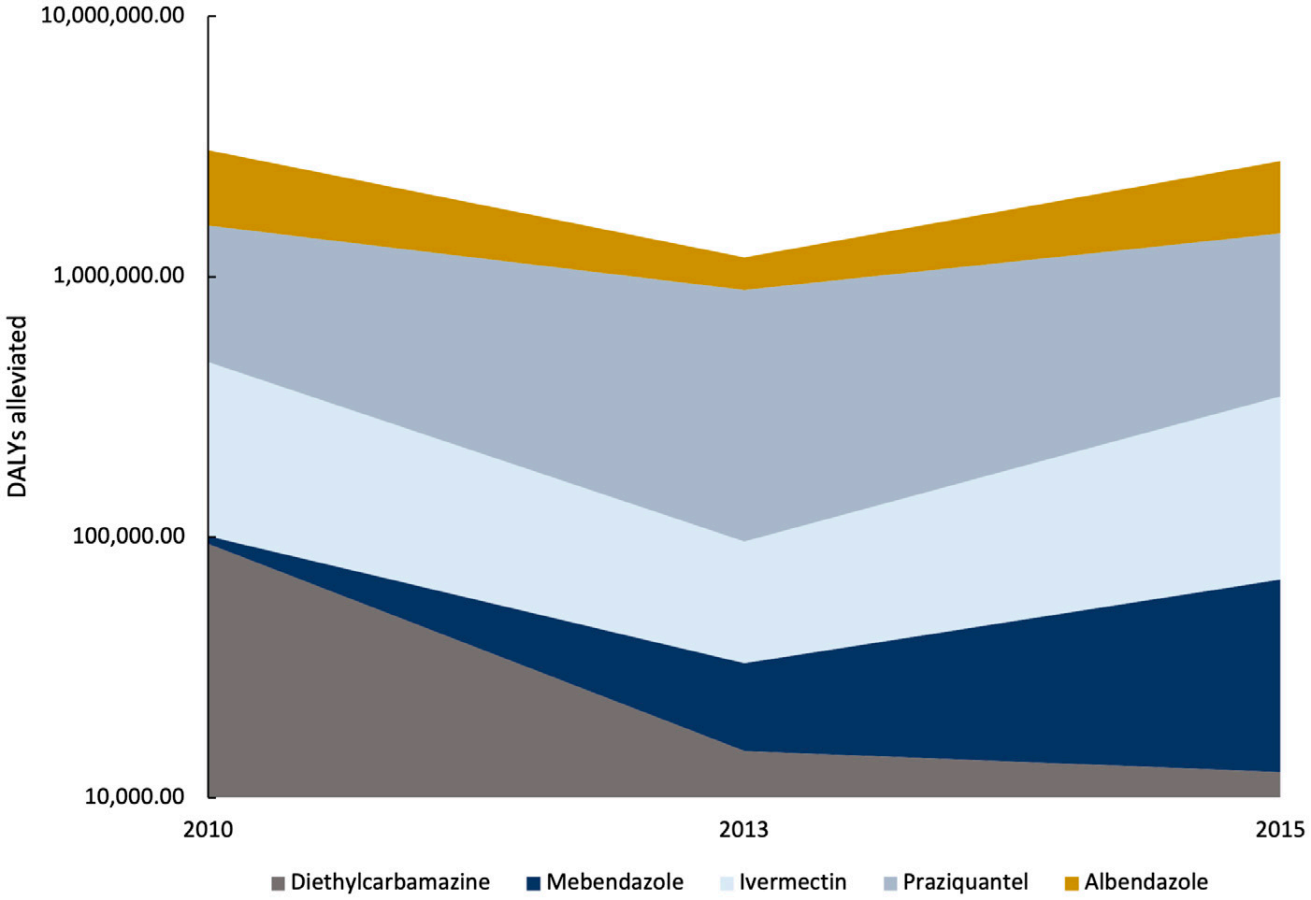
Drug impact vs. time. Data show the disability-adjusted life years (DALYs) alleviated by drug for all neglected tropical diseases, including schistosomiasis, soil-transmitted helminths, lymphatic filariasis, and onchocerciasis in 2010, 2013, and 2015.

Our models measure the impact of drugs used to treat NTDs. Albendazole, a key drug for STHs, has the largest impact out of all observed drugs because it is widely recommended and highly effective for all STHs besides whipworm; albendazole alleviates about 47.30% of the alleviated global burden of the NTDs in the models. Praziquantel for schistosomiasis also has a large impact. Figure 2 illustrates the impact of these drugs. Even with many highly effective drugs available, 65.79% of the burden of these diseases remains unalleviated: in 2015, our NTD models estimate that treatment averted 2,778,131.78 DALYs, leaving 8,121,497.75 DALYs accrued absent treatment globally.

**Figure 3.**
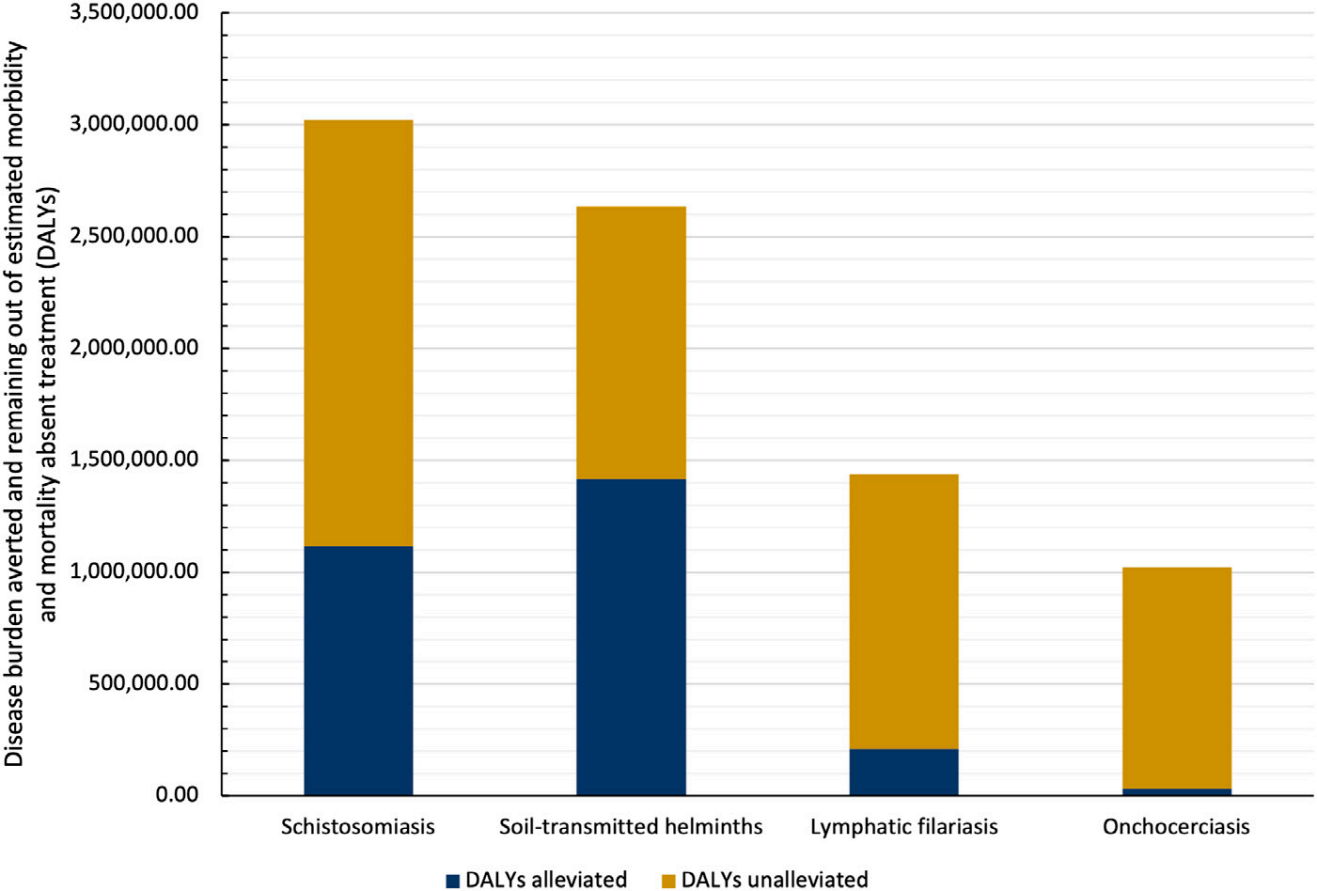
Unalleviated and alleviated disability-adjusted life years (DALYs) broken down by disease. Data show schistosomiasis, soil-transmitted helminths, lymphatic filariasis, and onchocerciasis percentage of DALYs averted and remaining out of the total estimated mortality and morbidity that would occur absent treatment in 2015.

Moreover, our models provide an overall picture of treatment impact on the six diseases observed. Together, treatments targeting STHs together averted 51.05% of the total DALYs averted from all NTD treatments, whereas onchocerciasis, lymphatic filariasis, and schistosomiasis medicines averted 1.18%, 7.56%, and 40.21%, respectively. Observing the global estimated need, or burden of disease in the absence of treatment, reveals that resources may not be allocated in the manner most efficient to eradicate these diseases. In fact, Figure 3 shows that although schistosomiasis presents the greatest overall need in 2015, only about one-third of it is alleviated with targeted treatment.

Finally, our models evaluate the impact of drugs aggregated by patent-holding companies. The 2015 model suggests drugs patented by GlaxoSmithKline and Merck avert about 57.31% of global NTD DALYs that are averted as a result of treatment. GlaxoSmithKline’s impact comes from its drug used to treat lymphatic filariasis and STHs, albendazole.

## DISCUSSION

Our models produce data that can provide states, nongovernmental organizations, and companies with the means of promoting new market strategies and innovative health policies to help achieve sustainable development goals that call to eliminate these NTDs by 2030. This is the first project of its kind that provides a common framework for evaluating treatment impact across a wide variety of interventions across several NTDs.†

Although many existing models try to predict the impact of treatment on the evolution of these diseases in a population, we estimate direct treatment impact in line with the other global health impact models (https://www.global-health-impact.org/new). With some modifications, the models can be rendered as part of traditional epidemiological models. Researchers can estimate the proportion of effectively treated individuals susceptible to reinfection, the number not effectively treated who transmit the disease to the larger susceptible population, the chance of transmission before treatment, and so forth. However, we avoid complicated mathematical modeling and do not make significant assumptions about patterns of change over time globally in the face of uncertainty. The advantage of our approach is that our models are simple and transparent and our results are not highly assumption driven.

We can improve estimates of treatment impact at the country level as further subnational data become available. Similarly, treatment effectiveness information can replace country-level drug efficacy studies that may overestimate drug effectiveness where available. We provide a summary of the data included in the 2015 model for countries where the diseases exist in Supplemental Information (data for other years are similar). Sensitivity analysis presented in Supplemental Information suggests, however, that the data limits do not have a large effect on our results.

Future research can improve upon our estimates by utilizing geographic, if not individual, data on treatment percentages where it is possible to acquire such data. The WHO does not currently provide data on the location of treatment sites and we were unable to secure this information upon request. However, some data may be available at the country level, for example, from DHIS2 health information systems.

These models may be refined in the future if researchers can separate the drugs’ impact based on different sequelae, that is, some sequelae are reversible whereas some can only be prevented, such as severe forms of elephantiasis. If it is possible to acquire data on the proportion of expected irreversible sequelae that are observed in treated populations, we could potentially figure out how many irreversible sequelae remain unaddressed by treatment and subtract that proportion from the estimated treatment impact, but we require data at the treatment level and currently lack the data to derive such estimates.

Researchers may also use different efficacy measures for different purposes. Right now, we use cure rates from treatment efficacy trials for onchocerciasis modeling, which does not accurately capture factors such as parasite burden and length of infection. Modeling utilizing egg reduction rate data may improve our estimates. Still, we incorporate the best existing data on a drug’s likely consequences into our models and conduct sensitivity analysis to determine how this affects results (see Supplemental Information).

Access to a framework that standardizes the health impact of NTDs and their interventions is critical in promoting equitable access to essential health care services by enabling policymakers to better understand, treat, and prevent NTDs. Existing models often try to predict time to elimination of these NTDs based on potential policies, but our models provide important information about impact before the diseases are eliminated. WHO-CHOICE, a model provided by the WHO, gives key decision makers information on cost-effectiveness and strategic planning.^28^ Our models provide important information on firms’ contributions but also aggregate information on drugs’ country- and disease-level effects essential for health system planning.

## CONCLUSION

There are several strategies currently deployed to combat NTDs around the world. National public health institutes and international organizations are contributing to the global control of NTDs through the development of laboratory surveillance tools and epidemiological methods to monitor program success.^29^ Pharmaceutical companies such as Pfizer, Merck, Novartis, and GlaxoSmithKline have donated millions of doses of drugs to diminish NTDs’ effects.^30^ There are also many public–private initiatives that aim to accelerate research and development of effective health tools such as diagnostics and vaccines to combat these diseases.^31^ Our results demonstrate that although we are making great strides in alleviating the burden of certain NTDs, pharmaceutical interventions may not be efficiently allocated (one can see this mismatch when comparing need versus treatment of global schistosomiasis cases, for instance). Although there are proven approaches to control the spread of NTDs, these diseases continue to cause a disproportionate amount of morbidity. Our models can help policymakers evaluate treatment access, set targets, and reduce the burden of NTD infection around the world.

## Supplemental files


Supplemental materials

## Footnotes

† We have also implemented a version of this model that reduces estimated impact by prevalence to account for the fact that treatment is primarily delivered via MDA, which is provided to almost all individuals in a given area (as only a percentage of the treated population will be infected with an NTD) to estimate only direct treatment. We provide this data on our website at global-health-impact.org
