# Supplementary material for: Global Health Impact: A Model to Alleviate the Burden and Expand Access to Treatment of Neglected Tropical Diseases
Source: Am J Trop Med Hyg. 2023 Feb 27;108(4):801–6. doi: 10.4269/ajtmh.21-0583 (PMC10077003; doi:10.4269/ajtmh.21-0583)
Supplement: Supplementary file 1 [file tpmd210583.SD1.pdf]

Supplementary Materials

Correspondence and requests for materials should be addressed to nhassoun@binghamton.edu.

This PDF files includes:

Example calculation
Data distribution
Sensitivity analysis
References
Tables S1 to S5
Figures S4 to S14

Example Calculation

Consider how we estimate drugs' impacts for Ethiopia. Schistosomiasis, onchocerciasis, and lymphatic filariasis are endemic in Ethiopia. Employing the WHO guidelines for treatment use in Tables S1 to S4 and Figures S4 to S14, we find that the population receives MDA1+T2; praziquantel is used to treat schistosomiasis, ivermectin is used to treat onchocerciasis, and ivermectin + albendazole is used to treat lymphatic filariasis.[1] We will first calculate the impact score for praziquantel. 210,340.92 DALYs were lost to schistosomiasis in Ethiopia in 2015.[2]

Estimated praziquantel efficacy in Ethiopia for schistosomiasis in 2015 is 94%.[3] Schistosomiasis treatment coverage was 28.80%.[4] We calculate impact for praziquantel on schistosomiasis in Ethiopia in 2015 in Equation S2:

$$I_{PZQ} = \frac{210{,}340.92 * 94\% * 28.80\%}{1 - (94\% * 28.80\%)} = 78{,}081.80$$

We calculate ivermectin's impact on onchocerciasis in the same way. 25,148.53 DALYs were lost to onchocerciasis in Ethiopia in 2015. Ivermectin efficacy in Ethiopia was 81%.[3] Onchocerciasis treatment

coverage was 64.23%.[4] For treatment to be effective, ivermectin must be administered every 6 months for the life span of the adult worms.[5] Adult worms can live in the nodules for approximately 15 years.[6] The overall impact formula for ivermectin's alleviated onchocerciasis DALYs is derived by Equation S3:

$$I_{IVM} = \frac{25{,}148.53 * 81\% * 64.23\%}{1 - (81\% * 64.23\%)} \div 2 \div 15 = 909.1$$

To calculate ivermectin + albendazole's impact on lymphatic filariasis, we used estimates of efficacy and treatment coverage from the same WHO region as Ethiopia because country-level data was not available in this case.[3] The overall impact formula for ivermectin's alleviated lymphatic filariasis DALYs is Equation S4:

$$I_{IVM + ALB} = \frac{1{,}168.19 * 29.43\% * 29.58\%}{1 - (29.43\% * 29.58\%)} = 111.4$$

We employ a similar methodology to derive impact scores for targeted NTD drugs in all endemic countries. We then aggregate all drug scores by country to get country-level impact estimates for all NTDs and by drug to get drug-level estimates for impacts both on individual NTDs and overall. We aggregate drug scores on individual diseases to get disease-level impact scores. Finally, we aggregate data on drugs' impacts by the originator company to provide company-level impact scores. We determine drug accreditation based on Table S5.

Data Distribution

Figure S1 shows that we have DALY data for 50 out of 54 countries for lymphatic filariasis (LF), 13 out of 53 countries for Onchocerciasis, 54 out of 69 countries for Schistosomiasis, and 22 out of 23 countries for Whipworm (under 5, 5-14 years, total years). The figure also shows we have DALY data for all 23 countries for Roundworm and Hookworm (under 5, 5-14 years, total years). Figure S2 shows that we

have country-level treatment percentages for all 54 countries for LF. We have country-level treatment percentage for 11 out of 23 countries for STH for Pre-SAC and 13 out of 23 countries for SAC, the rest use regional fall-back data. The figure also shows we have country-level treatment percentages for 36 out of 69 countries for Schist and 14 out of 53 countries for Onchocerciasis, 25 and 21 countries use regional-fall back data (respectively) and the rest use global estimates. Figure S3 shows we have country-level efficacy estimates for 21 out of 69 countries for Schist (praziquantel), 4 out of 53 countries for Onchocerciasis (ivermectin), 7 and 4 out of 54 countries for LF (for single dose of diethylcarbamazine, single dose of diethylcarbamazine + albendazole and albendazole+ivermectin, respectively), and we only have a few data points for the drugs used to treat Hookworm, Whipworm, and Roundworm out of the 23 countries in which they are endemic: 2 out of 23 countries for Hookworm (albendazole, mebendazole), 0, 1 and 3 out of 23 countries for Whipworm (mebendazole, albendazole, and ivermectin+albendazole respectively), and 0, 0 and 2 out of 23 countries for Roundworm (mebendazole, ivermectin+albendazole and albendazole). However, even in the later cases we have regional fallback data generated by country level data in the region but beyond the endemic area. Efficacy data was drawn from a review of the academic literature and is cited in the reference list below.[7-331]

Sensitivity analysis

We conducted sensitivity analyses to quantify uncertainty in our models. We ran a monte carlo simulation for 11 variables for 1,000 trials. For each trial, a value was randomly chosen from the assigned distribution. Company, drug, and disease rankings were then recalculated and any changes in rank were noted. In each test, if the parameter has a solution to the maximum likelihood estimator (MLE), then we use MLE; otherwise, we choose a particular mean based on our assumption with small variance (0.05). We define stability for an individual company or drug as follows: if the main box of the boxplot lies at 1 to -1, we will say the result is relatively stable. If no more than two rankings are unstable we classify our model as stable.

The y-axis indicates changes in company, drug, or disease ranks. Each number on the y-axis indicates the rank change e.g., 1 stands for a rank decrease by 1, -1 stands for rank increase by 1, 0 stands for not changing. The x-axis corresponds to company, drug, or disease for each year. For each company, drug, and disease combining all tests together, we processed the monte carlo test 1,000 times. So there are 1,000 new ranks for each company and drug. For example, Bayer might be ranked as follows in the different iterations: (4, 5, 6, 1, 8, 9, etc.), and then we compare each company's rank to its original rank to see whether its rank increased or decreased.

For each boxplot, the bold line in the middle represents the median, or 2nd quartile, and the box represents the 1st quartile to 3rd quartile. The upper whisker corresponds to the 3rd quantile + 1.5 * (3rd quartile - 1st quartile). Similarly, the lower whisker corresponds to the 1st quantile - 1.5 * (3rd quartile - 1st quartile). Any points above or below the whisker are outliers. We consider the upper whisker and lower whisker to be the maximum and minimum changed-rank in the 1,000 trials. The boxplot for each company represents the changed-rank.

Sensitivity analysis results

In 2010 all drugs had a stable ranking though albendazole's and mebendazole's rank has a tendency to move up in 50% of cases and praziquantel's and diethylcarbamazine's rank has a tendency to move down in 50% of cases. All companies held a stable ranking. Roundworm and whipworm's rank tended to move up in 50% of cases and schistosomiasis' and lymphatic filariasis' rank tended to move down in 50% of cases, though all stayed within the interquartile range. Although lymphatic filariasis' and roundworms' rank does exceed one positive interval change in the maximum case, the interquartile range remains within our acceptable bounds of stability. This is visualized in Figures S6, S9 and S12.

In 2013 all drugs had a stable ranking except for mebendazole and diethylcarbamazine; they changed rank by one positive interval and one negative interval, respectively (though the latter does exceed one positive

interval change in the maximum case it stayed within the interquartile range). All companies had a stable ranking. For diseases - onchocerciasis' rank tended to go up in 50% of cases, lymphatic filariasis' rank tended to go down in 50% of cases, although the later does exceed one positive interval change in the maximum case, both whipworm and lymphatic filariasis stayed in the interquartile range. See Figures S7, S10 and S13 for an illustration of these results.

In 2015 all drugs and diseases held a stable ranking. Merck went down one rank while Pfizer went up but overall the model was stable. This is shown in Figures S8, S11 and S14.

Test #1 assumption tested: Our model constrains the fallback data used to estimate treatment efficacy for NTDs to studies that took place before or during our model year. We tested different time constraints by removing all time constraints.

Test #2 assumption tested: In calculating lymphatic filariasis' impact score, the average regional treatment coverage of a drug is used as fallback data for that drug if treatment coverage data is not available for a country. When treatment coverage is not available for a given country, we assume it follows a beta distribution, with mean equal to global average and 0.05 variation.

Test #3 assumption tested: In calculating schistosomiasis's impact score, the average regional treatment coverage of a drug is used as fallback data for that drug if treatment coverage data is not available for a country. When treatment coverage is not available for a given country, we assume it follows a beta distribution, with mean equal to the global average and 0.05 variation.

Test #4 assumption tested: In calculating the impact score for soil-transmitted helminths, the average regional treatment coverage of a drug is used as fallback data for that drug if treatment coverage data is not available for a country. When efficacy data is not available for a given country, we assume it follows

beta distribution, with mean equal to the regional or global average and 0.05 variation.

Test #5 assumption tested: Assumption tested: In calculating lymphatic filariasis impact score, the average regional or global efficacy of a drug is used as fallback data for that drug if efficacy data is not available for a country. When efficacy data is not available for a given country, we assume it follows a beta distribution, with mean equal to regional or global average and 0.05 variation.

Test #6 assumption tested: In calculating schistosomiasis's impact score, the average regional or global efficacy of a drug is used as fallback data for that drug if efficacy data is not available for a country. When efficacy data is not available for a given country, we assume it follows a beta distribution, with mean equal to the regional or global average and 0.05 variation.

Test #7 assumption tested: In calculating whipworm's impact score, the average regional or global efficacy of a drug is used as fallback data for that drug if efficacy data is not available for a country. When efficacy data is not available for a given country, we assume it follows a beta distribution, with mean equal to the regional or global average and 0.05 variation.

Test #8 assumption tested: In calculating hookworm's impact score, the average regional or global efficacy of a drug is used as fallback data for that drug if efficacy data is not available for a country. When efficacy data is not available for a given country, we assume it follows beta distribution, with mean equal to the regional or global average and 0.05 variation.

Test #9 assumption tested: In calculating roundworm's impact score, the average regional or global efficacy of a drug is used as fallback data for that drug if efficacy data is not available for a country. When efficacy data is not available for a given country, we assume it follows beta distribution, with mean equal to the regional or global average and 0.05 variation.

Test #10 assumption tested: Our model uses the average regional and then global treatment coverage for an onchocerciasis drug as fallback data if that drug's treatment coverage data is not available for a given country. When treatment coverage is not available for a given country, we assume it follows a beta distribution, with mean equal to regional or global average and 0.05 variation.

Test #11 assumption tested: Our model uses the average regional and then global treatment efficacy for an onchocerciasis drug as fallback data if that drug's efficacy data is not available for a given country. When a drug's efficacy is not available for a given country, we assume the drug's efficacy follows a beta distribution, with mean equal to the regional or global average and 0.05 variation.

References

1. Crompton DWT, World Health Organization, 2006. Preventive chemotherapy in human helminthiasis. Coordinated use of anthelmintic drugs in control interventions: a manual for health professionals and programme managers. Geneva: World Health Organization. Available at: https://apps.who.int/iris/handle/10665/43545. Accessed April 1, 2022.
2. Global Burden of Disease Collaborative Network. Global Burden of Disease Study 2015 (GBD 2015) Disability-Adjusted Life Years and Healthy Life Expectancy 1990-2015. Seattle, United States of America: Institute for Health Metrics and Evaluation (IHME), 2015. **https://ghdx.healthdata.org/record/ihme-data/gbd-2015-dalys-hale-1990-2015** Accessed April 1, 2022.
3. Hassoun N, Friedman J, Cosler LE, 2020. Global Health Impact Data and Methodology. Available at: https://www.global-health-impact.org/static/docs/GHI%20Report.pdf. Accessed April 1, 2022.

4. World Health Organization, 2020 PCT Databank. Available at: https://www.who.int/neglected_diseases/preventive_chemotherapy/databank/en/. Accessed April 1, 2022.

5. Centers for Disease Control, 2010. Onchocerciasis: Treatment. Available at: https://www.cdc.gov/parasites/onchocerciasis/treatment.html. Accessed April 1, 2022.

6. Gyasi ME, Okonkwo ON, Tripathy K, 2020. Onchocerciasis. Treasure Island, FL: StatPearls Publishing.

7. Addiss DG, Beach MJ, Streit TG, Lutwick S, LeConte FH, Lafontant JG, Hightower AW, Lammie PJ, 1997. Randomised placebo-controlled comparison of ivermectin and albendazole alone and in combination for Wuchereria bancrofti microfilaraemia in Haitian children. *Lancet* 350: 480-484.

8. Andrade LD, Medeiros Z, Pires ML, Pimentel A, Rocha A, Figueredo-Silva J, 1995. Comparative efficacy of three different diethylcarbamazine regimens in lymphatic filariasis. *Trans R Soc Trop Med Hyg* 89: 319-321.

9. Beach MJ, Streit TG, Addiss DG, Prospere R, Roberts JM, Lammie PJ, 1999. Assessment of combined ivermectin and albendazole for treatment of intestinal helminth and Wuchereria bancrofti infections in Haitian school children. *Am J Trop Med Hyg* 60: 479–486.

10. Dreyer G, Addiss D, Noroes J, Amaral F, Rocha A, Coutinho A, 1996. Ultrasonographic assessment of the adulticidal efficacy of repeat high-dose ivermectin in bancroftian filariasis. *Trop Med Int Health* 1: 427–432.

11. Dreyer G, Addiss D, Williamson J, Norões J, 2006. Efficacy of co administered diethylcarbamazine and albendazole against adult Wuchereria bancrofti. *Trans R Soc Trop Med Hyg* 100: 1118–1125.

12. Dunyo SK, Nkrumah FK, Simonsen PE, 2000. A randomized double-blind placebo-controlled field trial of ivermectin and albendazole alone and in combination for the treatment of lymphatic filariasis in Ghana. *Trans R Soc Trop Med Hyg* 94: 205–211.

13. Dunyo SK, Simonsen PE, 2002. lvermectin and albendazole alone and in combination for the treatment of lymphatic filariasis in Ghana: follow-up after re-treatment with the combination. *Trans R Soc Trop Med Hyg* 96: 189–192.

14. Fischer P, Tukesiga E, Büttner DW, 1999. Long-Term Suppression of Mansonella streptocerca Microfilariae after Treatment with Ivermectin. *J Infect Dis* 180: 1403–1405.

15. Fox LM, Furness BW, Haser JK, Desire D, Brissau JM, Milord MD, Lafontant J, Lammie PJ, Beach MJ, 2005. Tolerance and efficacy of combined diethylcarbamazine and albendazole for treatment of Wuchereria bancrofti and intestinal helminth infections in Haitian children. *Am J Trop Med Hyg* 73: 115–21.

16. Gayen P, Nayak A, Saini P, Mukherjee N, Maitra S, Sarkar P, Babu SPS, 2012. A double-blind controlled field trial of doxycycline and albendazole in combination for the treatment of bancroftian filariasis in India. *Acta Tropica* 125: 150–156.

17. Setouhy ME, Ramzy RMR, Ahmed ES, Kandil AM, Hussain O, Farid HA, Helmy H, Weil GJ, 2004. A randomized clinical trial comparing single- and multi- dose combination therapy with diethylcarbamazine and albendazole for treatment of bancroftian filariasis. *Am J Trop Med Hyg* 70: 191–196.

18. Hoerauf A, Mand S, Fischer K, Kruppa T, Marfo-Debrekyei Y, Debrah AY, Pfarr KM, Adjei O, Buttner DW, 2003. Doxycycline as a novel strategy against bancroftian filariasis—depletion of Wolbachia endosymbionts from Wuchereria bancrofti and stop of microfilaria production. *Med Microbiol Immunol* 192: 211–216.

19. Hoti SL, Pani SP, Vanamail P, Athisaya M, Das LK, Das PK, 2010. Effect of a single dose of diethylcarbamazine, albendazole or both on the clearance of Wuchereria bancrofti microfilariae and antigenaemia among microfilaria carriers: A randomized trial. *Natl Med J India* 23: 72–76.

20. Zheng HJ, Piessens WF, Tao ZH, Cheng WF, Wang SH, Cheng SZ, Ye YM, Luo LF, Chen XR, Gan GB, 1991. Efficacy of ivermectin for control of microfilaremia recurring after treatment with diethylcarbamazine. I. Clinical and parasitological observations. *Am J Trop Med Hyg* 45: 168-174.

21. Ismail MM, Wei GJ, Jayasinghel KSA, Premaratne UN, Abeyewickremel W, Rajaratnam HN, Rezvi Sheriff'MH, Selvie Perera C, Dissanaike AS, 1996. Prolonged clearance of microfilaraemia in patients with bancroftian filariasis after multiple high doses of ivermectin or diethylcarbamazine. *Trans R Soc Trop Med Hyg* 90: 684–688.

22. Jayakody RL, De Silva CS, Weerasinghe WM, 1993. Treatment of bancroftian filariasis with albendazole: evaluation of efficacy and adverse reactions. *Trop Biomed* 10: 19–24.

23. Kazura J, Greenberg J, Perry R, Weil G, Day K, Alpers M, 1993. Comparison of single-dose diethylcarbamazine and ivermectin for treatment of bancroftian filariasis in Papua New Guinea. *Am Soc Trop Med Hyg* 49: 804-811.

24. Kshirsagar NA, et al., 2004. Safety, tolerability, efficacy and plasma concentrations of diethylcarbamazine and albendazole co-administration in a field study in an area endemic for lymphatic filariasis in India. *Trans R Soc Trop Med Hyg* 98: 205–217.

25. Noroes J, Dreyer G, Santos A, Mendes VG, Medeiros Z, Addiss D, 1997. Assessment of the efficacy of diethylcarbamazine on adult Wuchereria bancrofti in vivo. *Trans R Soc Trop Med Hyg* 91: 78-81.

26. Ottesen EA, Vijayasekaran V, Kumaraswami V, Pillai P, 1990. A controlled trial of ivermectin and diethylcarbamazine in lymphatic filariasis. *N Engl J Med* 322: 1113–1117.

27. Pani S, Subramanyam Reddy G, Das L, Vanamail P, Hoti SL, Ramesh J, Das PK, 2002. Tolerability and efficacy of single dose albendazole, diethylcarbamazine citrate (DEC) or co-administration of albendazole with DEC in the clearance of Wuchereria bancrofti in asymptomatic microfilaraemic volunteers in Pondicherry, South India: a hospital-based study. *Filaria J* 1: 1.

28. Richards FO Jr, Eberhard ML, Bryan RT, McNeeley DF, Lammie PJ, McNeeley MB, Bernard Y, Hightower AW, Spencer HC, 1991. Comparison of high dose ivermectin and diethylcarbamazine for activity against bancroftian filariasis in Haiti. *Am J Trop Med Hyg* 44: 3–10.

29. Shenoy RK, Dalia S, John A, Suma TK, Kumaraswami V, 1999. Treatment of the microfilaraemia of asymptomatic brugian filariasis with single doses of ivermectin, diethylcarbamazine or albendazole, in various combinations. *Ann Trop Med Parasitol* 93: 643–651.

30. Fischer P, Djuardi Y, Ismid IS, Rückert P, Bradley M, Supali T, 2003. Long-lasting reduction of Brugia timori microfilariae following a single dose of diethylcarbamazine combined with albendazole. *Trans R Soc Trop Med Hyg* 97: 446–448.

31. Shenoy RK, George LM, John A, Suma TK, Kumaraswami V, 1998. Treatment of microfilaraemia in asymptomatic brugian filariasis: the efficacy and safety of the combination of single doses of ivermectin and diethylcarbamazine. *Ann Trop Med Parasitol* 92: 579–585.

32. Shenoy RK, Suma TK, Rajan K, Kumaraswami V, 1998. Prevention of acute adenolymphangitis in brugian filariasis: comparison of the efficacy of ivermectin and diethylcarbamazine, each combined with local treatment of the affected limb. *Ann Trop Med Parasitol* 92: 587-594.

33. Supali T, Djuardi Y, Pfarr KM, Wibowo H, Taylor MJ, Hoerauf A, Houwing-Duistermaat JJ, Yazdanbakhsh M, Sartono E, 2008. Doxycycline treatment of Brugia malayi-infected persons reduces microfilaremia and adverse reactions after diethylcarbamazine and albendazole treatment. *Clin Infect Dis* 46: 1385–1393.

34. Taylor MJ, Makunde WH, McGarry HF, Turner JD, Mand S, Hoerauf A, 2005. Macrofilaricidal activity after doxycycline treatment of Wuchereria bancrofti: a double-blind, randomised placebo-controlled trial. *Lancet* 365: 2116–2121.

35. Wamae CN, Njenga SM, Ngugi BM, Mbui J, Njaanake HK, 2011. Evaluation of effectiveness of diethylcarbamazine/albendazole combination in reduction of Wuchereria bancrofti infection using multiple infection parameters. *Acta Trop* 120: 33–38.

36. Thomsen EK, et al, 2016. Efficacy, safety, and pharmacokinetics of coadministered diethylcarbamazine, albendazole, and ivermectin for treatment of bancroftian filariasis. *Clin Infect Dis* 62: 334–341.

37. Turner JD, Mand S, Debrah AY, Muehlfeld J, Pfarr K, McGarry HF, Adjei O, taylor MJ, Hoerauf A, 2006. A randomized, double-blind clinical trial of a 3-week course of doxycycline plus albendazole and ivermectin for the treatment of Wuchereria bancrofti infection. *Clin Infect Dis* 42: 1081-1089.

38. Ismail MM, Jayakody RL, Weil GJ, Fernando D, De Silva MS, De Silva GA, Balasooriya WK, 2001. Long-term efficacy of single-dose combinations of albendazole, ivermectin and diethylcarbamazine for the treatment of bancroftian filariasis. *Trans R Soc Trop Med Hyg* 95: 332–335.

39. Glaziou P, Moulia-Pelat JP, Nguyen LN, Chanteau S, Martin PM, Cartel JL, 1994. Double-blind controlled trial of a single dose of the combination ivermectin 400 micrograms/kg plus diethylcarbamazine 6 mg/kg for the treatment of bancroftian filariasis: results at six months. *Trans R Soc Trop Med Hyg* 88: 707–708.

40. Simonsen PE, Meyrowitach DW, Makunde WH, 1997. Bancroftian filariasis: long-term effect of the DEC provocative day test on microfilaraemia. *Trans R Soc Trop Med Hyg* 91: 290–293.

41. Fan PC, 1992. Diethylcarbamazine treatment of bancroftian and malayan filariasis with emphasis on side effects. *Ann Trop Med Parasitol* 86: 399–405.

42. Sabry M, Gamal H, el-Masry N, Kilpatrick ME, 1991. A placebo-controlled double-blind trial for the treatment of bancroftian filariasis with ivermectin or diethylcarbamazine. *Trans R Soc Trop Med Hyg* 85: 640–643.

43. Das PK, Ramaiah KD, Vanamail P, Pani SP, Yuvaraj J, Balarajan K, Bundy DA, 2001. Placebo-controlled community trial of four cycles of single-dose diethylcarbamazine or ivermectin against Wuchereria bancrofti infection and transmission in India. *Trans R Soc Trop Med Hyg* 95: 336–341.

44. Simonsen PE, Magesa SM, Dunyo SK, Malecela-Lazaro MN, Michael E, 2004. The effect of single dose ivermectin alone or in combination with albendazole on Wuchereria bancrofti infection in primary school children in Tanzania. *Trans R Soc Trop Med Hyg* 98: 462–472.

45. Addiss DG, Eberhard ML, Lammie PJ, McNeeley MB, Lee SH, McNeeley DF, Spencer HC, 1993. Comparative efficacy of clearing-dose and single high-dose ivermectin and diethylcarbamazine against Wuchereria bancrofti microfilaremia. *Am J Trop Med Hyg* 48: 178–185.

46. Dreyer G, Addiss D, Santos A, Figueredo-Silva J, Norões J, 1988. Direct assessment in vivo of the efficacy of combined single-dose ivermectin and diethylcarbamazine against adult Wuchereria bancrofti. *Trans R Soc Trop Med Hyg* 92: 219–222.

47. Dreyer G, et al., 1995. Treatment of bancroftian filariasis in Recife, Brazil: a two-year comparative study of the efficacy of single treatments with ivermectin or diethylcarbamazine. *Trans R Soc Trop Med Hyg* 89: 98–102.

48. Reddy GS, Vengatesvarlou N, Das PK, Vanamail P, Vijayan P, Kala S, Pani SP, 2000. Tolerability and efficacy of single-dose diethyl carbamazine (DEC) or ivermectin in the clearance of Wuchereria bancrofti microfilaraemia in Pondicherry. *South India Trop Med Int Health* 5: 779–785.

49. Chodakewitz J, 1995. Ivermectin and lymphatic filariasis: A clinical update. *Parasitol Today* 11: 233–235.

50. Dembele B, et al., 2010. Use of high-dose, twice-yearly albendazole and ivermectin to suppress Wuchereria bancrofti microfilarial levels. *Clin Infect Dis* 51: 1229–1235.

51. Bockarie MJ, Alexander NDE, Hyun P, Dimber Z, Bockarie F, Ibam E, Alpers MP, Kazura JW, 1998. Randomised community based trial of annual single-dose diethylcarbamazine with or without ivermectin against Wuchereria bancrofti infection in human beings and mosquitoes. *Lancet* 351: 1.

52. Ramaiah KD, Vanamail P, Pani SP, Yuvaraj J, Das PK. The effect of six rounds of single dose mass treatment with diethylcarbamazine or ivermectin on Wuchereria bancrofti infection and its implications for lymphatic filariasis elimination. *TM & IH* 7: 767–774.

53. Dunyo SK, Nkrumah FK, Simonsen PE, 2000. Single-dose treatment of Wuchereria bancrofti infections with ivermectin and albendazole alone or in combination: evaluation of the potential for control at 12 months after treatment. *Trans R Soc Trop Med Hyg* 94: 437–443.

54. Turner JD, et al., 2010. Macrofilaricidal activity after doxycycline only treatment of Onchocerca volvulus in an area of Loa loa Co-Endemicity: a randomized controlled trial. *PLoS Negl Trop Dis* 4: e660.

55. Hoerauf A, Mand S, Volkmann L, Büttner M, Marfo-Debrekyei Y, Taylor M, Adeji O, Buttner DW, 2003. Doxycycline in the treatment of human onchocerciasis: Kinetics of Wolbachia endobacteria reduction and of inhibition of embryogenesis in female Onchocerca worms. *Microbes Infect* 5: 261-273.

56. Awadzi K, Opoku NO, Attah SK, Lazdins-Helds J, Kuesel AC, 2014. A randomized, single-ascending-dose, ivermectin-controlled, double-blind study of moxidectin in Onchocerca volvulus infection. *PLoS Negl Trop Dis* 8: e2953.

57. Steel C, Lujan-Trangay A, Gonzalez-Peralta C, Zea-Flores G, Nutman TB, 1991. Immunologic responses to repeated ivermectin treatment in patients with onchocerciasis. *J Infect Dis* 164: 581–587.

58. Pacqué M, Muňoz B, Greene BM, Taylor HR, 1991. Community-based treatment of onchocerciasis with ivermectin: safety, efficacy, and acceptability of yearly treatment". *J Infect Dis* 163: 381–385.

59. White AT, Newland HS, Taylor HR, Erttmann KD, Keyvan-Larijani E, Nara A, Aziz MA, D'Anna SA, Williams PN, Greene BM, 1987. Controlled trial and dose-finding study of ivermectin for treatment of onchocerciasis. *J Infect Dis* 156: 463-470.

60. Kläger SL, Whitworth JA, Downham MD, 1996. Viability and fertility of adult Onchocerca volvulus after 6 years of treatment with ivermectin. *Trop Med Int Health* 1: 581–589.

61. Debrah AY, Mand S, Marfo-Debrekyei Y, Larbi J, Adjei O, Hoerauf A, 2006. Assessment of microfilarial loads in the skin of onchocerciasis patients after treatment with different regimens of doxycycline plus ivermectin. *Filaria J* 5: 1.

62. Aziz MA, Diallo S, Diop IM, Lariviere M, Porta M, 1982. Efficacy and tolerance of ivermectin in human onchocerciasis. *Lancet* 2: 171–173.

63. Fendt J, Hamm DM, Banla M, Schulz-Key H, Wolf H, Helling-Giese G, Heuschkel C, Soboslay PT, 2005. Chemokines in onchocerciasis patients after a single dose of ivermectin. *Clin Exp Immunol* 142: 318-326.

64. Awadzi K, Opoku NO, Attah SK, Addy ET, 1997. The safety and efficacy of amocarzine in African onchocerciasis and the influence of ivermectin on the clinical and parasitological response to treatment. *Ann Trop Med Parasitol* 91: 281–296.

65. Awadzi K, Edwards G, Duke BO, Opoku NO, Attah SK, Addy ET, Ardrey AE, Quartey BT, 2003. The co-administration of ivermectin and albendazole--safety, pharmacokinetics and efficacy against Onchocerca volvulus. *Ann Trop Med Parasitol* 97: 165–178.

66. Cline BL, Hernandez JL, Mather FJ, Bartholomew R, De Maza SN, Rodulfo S, Welborn CA, Eberhard ML, Convit J, 1992. Albendazole in the treatment of onchocerciasis: double-blind clinical trial in Venezuela. *Am J Trop Med Hyg* 47: 512–520.

67. Taylor HR, Langham ME, de Stahl EM, Figueroa LN, Beltranena F, 1980. Chemotherapy of onchocerciasis: a controlled clinical trial of topical diethylcarbamazine (DEC) in Guatemala. *Tropenmed Parasitol* 31: 357–364.

68. Taylor HR, Greene BM, Langham ME, 1980. Controlled clinical trial of oral and topical diethylcarbamazine in treatment of onchocerciasis. *Lancet* 1: 943–946.

69. Awadzi K, Attah SK, Addy ET, Opoku NO, Quartey BT, 1999. The effects of high-dose ivermectin regimens on Onchocerca volvulus in onchocerciasis patients. *Trans R Soc Trop Med Hyg* 93: 189–194.

70. Newland HS, White AT, Greene BM, D'Anna SA, Keyvan-Larijani E, Aziz MA, Williams PN, Taylor HR, 1988. Effect of single-dose ivermectin therapy on human Onchocerca volvulus infection with onchocercal ocular involvement. *Br J Ophthalmol* 72: 561–529.

71. Mössinger J, Schulz-Key H, Dietz K, 1988. Emergence of Onchocerca volvulus microfilariae from skin snips before and after treatment of patients with ivermectin. *Trop Med Parasitol* 39: 313–316.

72. Kebede A, Taticheff S, Bulto T, Workneh W, Tilahun D, 1993. Effect of ivermectin treatment on microfilarial load in patients with Onchocerca volvulus in Bebeka, Ethiopia. *Ethiop Med J* 31: 127–135.

73. Richards FO Jr, Flores ZR, Duke BOL, 1989. Dynamics of microfilariae of Onchocerca volvulus over the first 72 hours after treatment with ivermectin. *Trop Med Parasitol* 40: 299-303.

74. Albiez EJ, Newland HS, White AT, Kaiser A, Greene BM, Taylor HR, Buttner DW, 1988. Chemotherapy of onchocerciasis with high doses of diethylcarbamazine or a single dose of ivermectin: microfilaria levels and side effects. *Trop Med Parasitol* 39: 19-24.

75. Taylor HR, Murphy RP, Newland HS, White AT, D'Anna SA, Keyvan-Larijani E, Aziz MA, Cupp EW, Greene BM, 1986. Treatment of onchocerciasis. The ocular effects of ivermectin and diethylcarbamazine. *Arch Ophthalmol* 104: 863–870.

76. Greene BM, Dukuly ZD, Muñoz B, White AT, Pacqué M, Taylor HR, 1991. A comparison of 6, 12, and 24-monthly dosing with ivermectin for treatment of onchocerciasis. *J Infect Dis* 163: 376–380.

77. Duke BO, Zea-Flores G, Castro J, Cupp EW, Munoz B, 1991. Comparison of the effects of a single dose and of four six-monthly doses of ivermectin on adult Onchocerca volvulus. *Am J Trop Med Hyg* 45: 132–137.

78. Awadzi K, Dadzie KY, Schulz-Key H, Gilles HM, Fulford AJ, Aziz MA, 1986. The chemotherapy of onchocerciasis. XI. A double-blind comparative study of ivermectin, diethylcarbamazine and placebo in human onchocerciasis in northern Ghana. *Ann Trop Med Parasitol* 80: 433-442.

79. Diallo S, et al., 1986. A double-blind comparison of the efficacy and safety of ivermectin and diethylcarbamazine in a placebo controlled study of Senegalese patients with onchocerciasis. *Trans R Soc Trop Med Hyg* 80: 927–934.

80. Whitworth JAG, Downham MD, Lahai G, Maude GH, 1996. A community trial of ivermectin for onchocerciasis in Sierra Leone: compliance and parasitological profiles after three and a half years of intervention. *Trop Med Int Health* 1: 52–58.

81. Anderson J, Fuglsang H, 1978. Further studies on the treatment of ocular onchocerciasis with diethylcarbamazine and suramin. *Br J Ophthalmol* 62: 450–457.

82. Awadzi K, Hero M, Opoku NO, Addy ET, Büttner DW, Ginger CD, 1995. The chemotherapy of onchocerciasis XVIII. Aspects of treatment with suramin. *Trop Med Parasitol* 46: 19–26.

83. Burch TA, Ashburn LL, 1951. Experimental therapy of onchocerciasis with suramin and hetrazan; results of a three-year study. *Am J Trop Med Hyg* 31: 617–623.

84. Burch TA, 1949. Experimental Therapy of Onchocerciasis with Suramin and Hatrazan. Boletin de la Oficina Sanitaria Panamericana. *Pan American Sanitary Bureau* 28: 233–248.

85. Chijoke CP, Umeh RE, Mbah AU, Nwonu P, Fleckenstein LL, Okonkwo PO, 1998. Clinical pharmacokinetics of suramin in patients with onchocerciasis. *Eur J Clin Pharmacol* 54: 249–251.

86. Anderson J, Fuglsang H, de C Marshall T F, 1976. Effects of suramin on ocular onchocerciasis. *Tropenmed Parasitol* 27: 279–296.

87. Wolf H, Schulz-Key H, Albiez EJ, Geister R, Büttner DW, 1980. Analysis of Enzymatically Isolated Adults of Onchocerca volvulus after Treatment of Patients with Suramin or Metrifonate. *Tropenmed Parasitol* 31: 143–148.

88. Duke BO, Vincelette J, Moore PJ, 1976. The population dynamics of Onchocerca volvulus microfilariae during treatment with suramin and diethylcarbamazine. *Tropenmed Parasitol* 27: 133–144.

89. Lariviere M, et al., 1985. Double-blind study of ivermectin and diethylcarbamazine in African onchocerciasis patients with ocular involvement. *Lancet* 27: 174–177.

90. Awadzi K, Gilles HM, 1980. The chemotherapy of onchocerciasis III A comparative study of diethylcarbamazine (DEC) and metrifonate. *Ann Trop Med Parasitol* 74: 199-210.

91. Duke BO, 1968. The effects of drugs on Onchocerca volvulus. 3. Trials of suramin at different dosages and a comparison of the brands Antrypol, Moranyl and Naganol. *Bull World Health Organ* 39: 157–167.

92. Hoerauf A, et al., 2008. Wolbachia endobacteria depletion by doxycycline as antifilarial therapy has macrofilaricidal activity in onchocerciasis: a randomized placebo-controlled study. *Med Microbiol Immunol* 197: 295–311.

93. Schulz-Key H, Karam M, Prost A, 1985. Suramin in the treatment of onchocerciasis: the efficacy of low doses on the parasite in an area with vector control. *Trop Med Parasitol* 36: 244–248.

94. Coulibaly YI, et al, 2009. A randomized trial of doxycycline for Mansonella perstans infection. *N Engl J Med* 361: 1448-1458.

95. Masud H, Qureshi T, Dukley M, 2009. Effects of Ivermectin with and without Doxycycline on Clinical Symptoms of Onchocerciasis. *J Coll Physicians Surg Pak* 19: 34–38.

96. Richards FO Jr, et al., 2007. No depletion of Wolbachia from Onchocerca volvulus after a short course of rifampin and/or azithromycin. *Am J Trop Med Hyg* 77: 878–882.

97. Hoerauf A, Marfo-Debrekyei Y, Büttner M, Debrah AY, Konadu P, Mand S, Adjei O, Buttner DW, 2008. Effects of 6-week azithromycin treatment on the Wolbachia endobacteria of Onchocerca volvulus. *Parasitol Res* 103: 279-286.

98. Hoerauf A, Mand S, Adjei O, Fleischer B, Büttner DW, 2001. Depletion of wolbachia endobacteria in Onchocerca volvulus by doxycycline and microfilaridermia after ivermectin treatment. *Lancet* 357: 1415–1416.

99. Banic DM, Calvão-Brito RHS, Marchon-Silva V, Schuertez JC, de Lima Pinheiro LR, Alves MDC, Têva A, Maia-Herzog M, 2009. Impact of 3 years ivermectin treatment on onchocerciasis in Yanomami communities in the Brazilian Amazon. *Acta Trop* 112: 125–130.

100. Pion SD , Grout L, Kamgno J, Nana-Djeunga H, Boussinesq M, 2011. Individual host factors associated with Onchocerca volvulus microfilarial densities 15, 80 and 180 days after a first dose of ivermectin. *Acta tropica* 120: 91–99.

101. Boussinesq M, Chippaux JP, Ernould JC, 1993. Parasitological efficacy of repeated treatments with Ivermectin in an Onchocerciasis focus in North Cameroon. *Bull Soc Pathol Exot* 86: 112–115.

102. Tamarozzi F, Tendongfor N, Enyong PA, Esum M, Faragher B, Wanji S, Taylor MJ, 2012. Long term impact of large scale community-directed delivery of doxycycline for the treatment of onchocerciasis. *Parasit Vectors* 5: 53.

103. Hoerauf A, et al., 2009. Efficacy of 5-week doxycycline treatment on adult Onchocerca volvulus. *Parasitol Res* 104: 437–447.

104. Specht S, Mand S, Marfo-Debrekyei Y, Debrah AY, Konadu P, Adjei O, Buttner DW, Hoerauf A, 2008. Efficacy of 2- and 4- week of Rifampicin treatment on the Wolbachia of Onchocerca volvulus. *Parasitol Res* 103: 1303–1309.

105. Oyibo WA, Fagbenro-Beyioku AF, 2003. Effect of repeated community-based ivermectin treatment on the intensity of onchocerciasis in Nigeria. *Rural Remote Health* 3: 211.

106. Walker M, Specht S, Churcher TS, Heorauf A, Taylor MJ, Basanex MG, 2014. Therapeutic efficacy and macrofilaricidal activity of doxycycline for the treatment of river blindness. *Clin Infect Dis* 60: 1199–1207.

107. Plaisier AP, Alley ES, Boatin BA, Van Oortmarssen GJ, Remme H, de Vlas SJ, Bonneux L, Habbema DF, 1995. Irreversible effects of Ivermectin on adult parasites in onchocerciasis patients in the onchocerciasis control programme in West Africa. *J Infect Dis* 172: 204–210.

108. Rodriguez-Perez, MA, Rodriguez MH, Margeli-Perez HM, 1995. Effect of semiannual treatments of ivermectin on the prevalence and intensity of onchocerca volvulus skin infection, ocular lesions, and infectivity of simulium ochraceum populations in Southern Mexico. *Am J Trop Med Hyg* 52: 429-434.

109. Ndyomugyenyi R, 2004. The impact of ivermectin treatment alone and when in parallel with Simulium neavei elimination on onchocerciasis in Uganda. *J Trop Med Int Health* 9: 882-886.

110. Opoku N, et al., 2013. Efficacy of a single oral dose of 8 mg Moxidectin vs. 150 ug/kg ivermectin in ochocerca volvulus infection: results of a randomized, double-blind single dose phase 3 study in areas without mass treatment with ivermectin in Liberia, Ghana and DRC. *Trop Med Int Health* 18: 52-107.

111. Osei-Atweneboana M, Eng JKL, Boakye DA, Gyapong JO, Prichard RK, 2007. Prevalence and intensity of Onchocerca volvulus infection and efficacy of ivermectin in endemic communities in Ghana: a two-phase epidemiological study. *Lancet* 369: 2021–2029.

112. Dadzie KY, Remme J, Alley ES, de Sole G, 1990. Changes in ocular onchocerciasis four and twelve months after community-based treatment with ivermectin in a holoendemic onchocerciasis focus. *Trans R Soc Trop Med Hyg* 84: 103–108.

113. Rodger FC, 1958. Comparison of the Effect Upon Onchocerciasis of Five Drugs and Selection of the One Best Able to Prevent Ocular Complications. *Trans R Soc Trop Med Hyg* 52: 462–467.

114. Ayele T, 1984. Preliminary clinical trial of oral oxamniquine in the treatment of Schistosoma mansoni in Ethiopia. *East Afr Med J* 61: 632–636.

115. Barakat R, Elmorshedy H, Fenwick A, 2005. Efficacy of myrrh in the treatment of human Schistosomiasis mansoni. *Am J Trop Med Hyg* 73: 365–367.

116. Borrmann S, Szlezák N, Faucher JF, Matsiegui PB, Neubauer R, Binder RK, Lell B, Kremser PG, 2001. Artesunate and praziquantel for the treatment of Schistosoma haematobium infections: a double-blind, randomized, placebo-controlled study. *J Infect Dis* 184: 1363–1366.

117. Botros S, Sayed H, Amer N, El-Ghannam M, Bennett JL, Day TA, 2005. Current status of sensitivity to praziquantel in a focus of potential drug resistance in Egypt. *Int J Parasitol* 35: 787–791.

118. Branchini ML, Pedro Rde J, Dias LC, Deberaldini ER, 1982. Double-blind clinical trial comparing praziquantel with oxamniquine in the treatment of patients with schistosomiasis mansoni. *Rev Inst Med Trop Sao Paulo* 24: 315–321.

119. Senghor, Bruno, Diaw OT, Doucoure S, Sylla SN, Seye M, Talla I, Ba CT, Diallo A, Sokhna C, 2015. Efficacy of praziquantel against urinary schistosomiasis and reinfection in Senegalese school children where there is a single well-defined transmission period. *Parasit Vectors* 8: 1–11.

120. Stete K, et al., 2012. Dynamics of Schistosoma haematobium egg output and associated infection parameters following treatment with praziquantel in school-aged children. *Parasit Vectors* 5: 298.

121. Creasey AM, Taylor P, Thomas JE, 1986. Dosage trial of a combination of oxamniquine and praziquantel in the treatment of schistosomiasis in Zimbabwean schoolchildren. *Cent Afr J Med* 32: 165–167.

122. da Cunha AS, Pedrosa RC, 1986. Double-blind therapeutical evaluation based on the quantitative oogram technique, comparing praziquantel and oxamniquine in human schistosomiasis mansoni. *Rev Inst Med Trop Sao Paulo* 28: 337–351.

123. da Silva LC, et al., 1986. A randomized trial for the study of the elective surgical treatment of portal hypertension in mansonic schistosomiasis. *Ann Surg* 204: 148–153.

124. Davis A, Biles JE, Ulrich AM, 1979. Initial experiences with praziquantel in the treatment of human infections due to Schistosoma haematobium. *Bull World Health Organ* 57: 773–779.

125. de V Clarke V, Blair DM, Weber MC, Garnett PA, 1976. Dose-finding trials of oral oxamniquine in Rhodesia. *S Afr Med J* 50: 1867–1871.

126. De Clercq D, Vercruysse J, Verlé P, Kongs A, Diop M, 2000. What is the effect of combining artesunate and praziquantel in the treatment of Schistosoma mansoni infections? *Trop Med Int Health* 5: 744–746.

127. de Jonge N, Schommer G, Feldmeier H, Krijger FW, Dafalla AA, Bienzle U, Deelder AM, 1990. Mixed Schistosoma haematobium and S. mansoni infection: effect of different treatments on the serum level of circulating anodic antigen (CAA). *Acta Trop* 48: 25–35.

128. Queiroz LC, Drummond SC, Matos ML, Paiva MB, Batista TS, Kansaon AZ, de F Antunes CM, Lambertucci JR, 2010. Comparative randomised trial of high and conventional doses of praziquantel in the treatment of schistosomiasis mansoni. *Mem Inst Oswaldo Cruz* 105*:* 445–448.

129. Stelma FF, Sall S, Daff B, Sow S, Niang M, Gryseels B, 1997. Oxamniquine cures Schistosoma mansoni infection in a focus in which cure rates with praziquantel are unusually low. *J Infect Dis* 176: 304–307.

130. Ferrari ML, Coelho PM, Antunes CM, Tavares CA, da Cunha AS, 2003. Efficacy of oxamniquine and praziquantel in the treatment of Schistosoma mansoni infection: a controlled trial. *Bull World Health Organ* 81: 190–196.

131. Giboda M, et al., 1992. Efficacy of praziquantel treatment of schistosomiasis in a non-endemic country: a follow-up of parasitological, clinical and immunological parameters. *J Hyg Epidemiol Microbiol Immunol* 36: 346–355.

132. Gryseels B, Nkulikyinka L, 1989. Two-year follow-up of Schistosoma mansoni infection and morbidity after treatment with different regimens of oxamniquine and praziquantel. *Trans R Soc Trop Med Hyg* 83: 219–228.

133. Guisse F, Polman K, Stelma FF, Mbaye A, Talla I, Niang M, Deelder AM, Ndir O, Gryseels B, 1997. Therapeutic evaluation of two different dose regimens of praziquantel in a recent Schistosoma mansoni focus in Northern Senegal. *Am J Trop Med Hyg* 56: 511–514.

134. Gupta KK, 1984. Schistosoma mansoni treatment with oral oxamniquine in Zambia. *East Afr Med J* 61: 641–644.

135. Hou XY, McManus DP, Gray DJ, Balen J, Luo XS, He YK, Ellis M, Williams GM, Li YS, 2008. A randomized, double-blind, placebo-controlled trial of safety and efficacy of combined praziquantel and artemether treatment for acute schistosomiasis japonica in China. *Bull World Health Organ* 86: 788–795.

136. Ibrahim AM, 1980. Evaluation of oxamniquine in the treatment of S. mansoni infection among Sudanese patients. *East Afr Med J* 57: 566–573.

137. Inyang-Etoh PC, Ejezie GC, Useh MF, Inyang-Etoh EC, 2009. Efficacy of a combination of praziquantel and artesunate in the treatment of urinary schistosomiasis in Nigeria. *Trans R Soc Trop Med Hyg* 103: 38–44.

138. Kabatereine NB, Kemijumbi J, Ouma JH, Sturrock RF, Butterworth AE, Madsen H, Ornbjerg N, Dunne DW, Vennervald BJ, 2003. Efficacy and side effects of praziquantel treatment in a highly endemic Schistosoma mansoni focus at Lake Albert, Uganda. *Trans R Soc Trop Med Hyg* 97: 599–603.

139. Kardaman MW, Amin MA, Fenwick A, Cheesmond AK, Dixon HG, 1983. A field trial using praziquantel (Biltricide) to treat Schistosoma mansoni and Schistosoma haematobium infection in Gezira, Sudan. *Ann Trop Med Parasitol* 77: 297–304.

140. Kilpatrick ME, El Masry NA, Bassily S, Farid Z, 1982. Oxamniquine versus niridazole for treatment of uncomplicated Schistosoma mansoni infection. *Am J Trop Med Hyg* 31: 1164–1167.

141. Lambertucci JR, Greco DB, Pedrosa ERP, da Costa Rocha MO, Salazar HM, De Lima DPA, 1982. Double blind trial with oxamniquine in chronic schistosomiasis mansoni. *Trans Roy Soc Trop Med Hyg* 76: 751–755.

142. Li YS, Chen HG, He HB, Hou XY, Ellis M, McManus DP, 2005. A double-blind field trial on the effects of artemether on Schistosoma japonicum infection in a highly endemic focus in southern China. *Acta Trop* 96: 184–190.

143. Metwally A, Bennett J, Botros S, Ebeid F, el attar Gel D, 1995. Impact of drug dosage and brand on bioavailability and efficacy of praziquantel. *Pharmacol Res* 31: 53–59.

144. N'Goran EK, Utzinger J, Gnaka HN, Yapi A, N'Guessan NA, Kigbafori SD, Lengeler C, Chollet J, Shuhua X, Tanner M, 2003. Randomized, double-blind, placebo-controlled trial of oral artemether for the prevention of patent Schistosoma haematobium infections. *Am J Trop Med Hyg* 68: 24–32.

145. Olliaro PL, et al., 2011. A multicentre randomized controlled trial of the efficacy and safety of single-dose praziquantel at 40 mg/kg vs. 60 mg/kg for treating intestinal schistosomiasis in the Philippines, Mauritania, Tanzania and Brazil. *PLoS Negl Trop Dis* 5: e1165.

146. Ouldabdallahi M, Ousmane B, Ouldbezeid M, Mamadou D, Konaté L, Chitsulo L, 2013. Comparaison de l'efficacité thérapeutique et de la tolérance du praziquantel administré en prise unique à la dose de 40 versus 60 mg/kg pour le traitement de la bilharziose urinaire en Mauritanie [Comparison of the efficacy and safety of praziquantel administered in single dose of 40 versus 60 mg/kg for treating urinary schistosomiasis in Mauritania]. *Bull Soc Pathol Exot* 106: 167–169.

147. Pugh RN, Teesdale CH, 1983. Single dose oral treatment in urinary schistosomiasis: a double blind trial. *Br Med J (Clin Res Ed)* 286: 429–432.

148. Raso G, et al., 2004. Efficacy and Side Effects of Praziquantel against Schistosoma Mansoni in a Community of Western Cˆote D'Ivoire. *Trans R Soc Trop Med Hyg* 98: 18–27.

149. Rezende GL, 1985. Survey on the clinical results achieved in Brazil comparing praziquantel and oxamniquine in the treatment of S. mansoni schistosomiasis. *Rev Inst Med Trop* 27: 328–368.

150. Saathoff E, Olsen A, Magnussen P, Kvalsvig JD, Becker W, Appleton CC, 2004. Patterns of Schistosoma haematobium infection, impact of praziquantel treatment and re-infection after treatment in a cohort of schoolchildren from rural KwaZulu-Natal/South Africa. *BMC Infect Dis* 4: 40.

151. da Silva LC, Zeitune JM, Rosa-Eid LM, Lima DM, Antonelli RH, Christo CH, Saez-Alquezar A, Carboni A de C, 1986. Treatment of patients with schistosomiasis mansoni: a double blind

clinical trial comparing praziquantel with oxamniquine. *Rev Inst Med Trop Sao Paulo* 28: 174–180.

152. Taddese K, Zein ZA, 1988. Comparison between the efficacy of oxamniquine and praziquantel in the treatment of Schistosoma mansoni infections on a sugar estate in Ethiopia. *Ann Trop Med Parasitol* 82: 175–180.

153. Tweyongyere R, et al., 2009. Effect of praziquantel treatment of Schistosoma mansoni during pregnancy on intensity of infection and antibody responses to schistosome antigens: results of a randomised, placebo-controlled trial. *BMC Infect Dis* 9: 32.

154. Zwingenberger K, Queiroz JA, Poggensee U, Alencar JE, Valdegunas J, Esmeralda F, Feldmeier H, 1987. Efficacy of oxamniquine, praziquantel and a combination of both drugs in schistosomiasis mansoni in Brazil. *Rev Inst Med Trop* 29: 305–311.

155. Utzinger J, N'Goran EK, N'Dri A, Lengeler C, Tanner M, 2000. Efficacy of praziquantel against Schistosoma mansoni with particular consideration for intensity of infection. *Trop Med Int Health* 5: 771–778.

156. Utzinger J, N'Goran EK, N'Dri A, Lengeler C, Xiao S, Tanner M, 2000. Oral artemether for prevention of Schistosoma mansoni infection: randomised controlled trial. *Lancet* 355: 1320–1325.

157. Mohamed AA, Mahgoub HM, Magzoub M, Gasim GI, Eldein WN, Ahmed Ael A, Adam I, 2009. Artesunate plus sulfadoxine/pyrimethamine versus praziquantel in the treatment of Schistosoma mansoni in eastern Sudan. *Trans R Soc Trop Med Hyg* 103: 1062–1064.

158. Navaratnam AMD, Sousa-Figueiredo JC, Stothard JR, Kabatereine NB, Fenwick A, Mutumba-Nakalembe MJ, 2012. Efficacy of praziquantel syrup versus crushed praziquantel tablets in the treatment of intestinal schistosomiasis in Ugandan preschool children, with observation on compliance and safety. *Trans R Soc Trop Med Hyg* 106: 400–407.

159. Obonyo CO, Muok EM, Mwinzi PN, 2010. Efficacy of artesunate with sulfalene plus pyrimethamine versus praziquantel for treatment of Schistosoma mansoni in Kenyan children: an open-label randomised controlled trial. *Lancet Infect Dis* 10: 603–611.

160. Tchuenté LA, Shaw DJ, Polla L, Cioli D, Vercruysse J, 2004. Efficacy of praziquantel against Schistosoma haematobium infection in children. *Am J Trop Med Hyg* 71: 778–782.

161. King CH, Muchiri EM, Mungai P, Ouma JH, Kadzo H, Magak P, Koech DK, 2002. Randomized comparison of low-dose versus standard-dose praziquantel therapy in treatment of urinary tract morbidity due to Schistosoma haema tobium infection. *Am J Trop Med Hyg* 66: 725–730.

162. Garba A, Lamine MS, Djibo A, Tahirou A, Aouami MA, Alfari A, Phillips AE, Fenwick A, Utzinger J, 2013. Safety and efficacy of praziquantel syrup (Epiquantel®) against Schistosoma haematobium and Schistosoma mansoni in preschool-aged children in Niger. *Acta Trop* 128: 318–325.

163. Keiser J, N'Guessan NA, Adoubryn KD, Silué KD, Vounatsou P, Hatz C, Utzinger J, N'Goran EK, 2010. Efficacy and safety of mefloquine, artesunate, mefloquine-artesunate, and praziquantel against Schistosoma haematobium: randomized, exploratory open-label trial. *Clin Infect Dis* 50: 1205–1213.

164. Keiser J, Silué KD, Adiossan LK, N'Guessan NA, Monsan N, Utzinger J, N'Goram EK, 2014. Praziquantel, mefloquine-praziquantel, and mefloquine-artesunate-praziquantel against Schistosoma haematobium: a randomized, exploratory, open-label trial. *PLoS Negl Trop Dis* 8: e2975.

165. Mutapi F, Rujeni N, Bourke C, Mitchell K, Appleby L, Nausch N, Midzi N, Mduluza T, 2011. Schistosoma haematobium treatment in 1-5 year old children: safety and efficacy of the antihelminthic drug praziquantel. *PLoS Negl Trop Dis* 5: e1143.

166. Nalugwa A, Nuwaha F, Tukahebwa EM, Olsen A, 2015. Single versus double dose praziquantel comparison on efficacy and Schistosoma mansoni re-Infection in preschool-age children in Uganda: a randomized controlled trial. *PLoS Negl Trop Dis* 9: e0003796.

167. N'Goran EK, Gnaka HN, Tanner M, Utzinger J, 2003. Efficacy and side-effects of two praziquantel treatments against Schistosoma haematobium infection, among schoolchildren from Côte d'Ivoire. *Ann Trop Med Parasitol* 97: 37–51.

168. Al-Aska AK, Al-Mofleh IA, Al-Rashed R, Hafez MA, Al- Nozha M, Abu-Aisha H, Al-Balla SR, Taha A, 1990. Praziquantel, oxamniquine, and metrifonate in the treatment of schistosomiasis in Riyadh. *Ann Saudi Med* 10: 296–298.

169. Ayele T, 1986. Preliminary clinical trial of oral oxamniquine in the treatment of Schistosoma mansoni in children in Ethiopia. *East Afr Med J* 63: 291–294.

170. Fernandes P, Oliveira CC, 1986. Efficacy of two regimes of praziquantel versus oxamniquine [Estudo comparativo da eficacia do praziquantel, em dois esquemas posologicos, e da oxaminiquina no tratamento da esquistossomose mansonica]. *Folha Medica* 93: 389–393.

171. Homeida MM, Eltom IA, Sulaiman SM, Ali HM, Bennett JL, 1989. Tolerance of two brands of praziquantel. *Lancet* 334: 391.

172. Katz N, Rocha RS, Chaves A, 1981. Clinical trials with praziquantel in Schistosomiasis mansoni. *Rev Inst Med Trop Sao Paulo* 23: 72–28.

173. Katz N, Rocha RS, 1982. Double-blind clinical trial comparing praziquantel with oxamniquine in Schistosomiasis mansoni. *Rev Inst Med Trop Sao Paulo* 24: 310–314.

174. Omer AH, 1981. Praziquantel in the treatment of mixed S. haematobium and S. mansoni infections. *Arzneimittelforschung* 31: 605–608.

175. Rugemalila JB, Asila J, Chimbe A, 1984. Randomized comparative trials of single doses of the newer antischistosomal drugs at Mwanza, Tanzania. I. Praziquantel and oxamniquine for the treatment of Schistosomiasis mansoni. *J Trop Med Hyg* 87: 231–235.

176. Taylor P, Murare HM, Manomano K, 1988. Efficacy of low doses of praziquantel for Schistosoma mansoni and S. haematobium. *J Trop Med Hyg* 91: 13–17.

177. Teesdale CH, Chitsulo L, Pugh RN, 1984. Oxamniquine dosage in Malawi. *East Afr Med J* 61: 40–44.

178. Zhang Y et al., 2007. Parasitological impact of 2-year preventive chemotherapy on schistosomiasis and soil-transmitted helminthiasis in Uganda. *BMC Med* 5: 27.

179. Tohon ZB, Mainassara HB, Garba A, Mahamane AE, Bosqué-Oliva E, Ibrahim ML, Duchemin JB, Chanteau S, Boisier P, 2008. Controlling schistosomiasis: significant decrease of anaemia prevalence one year after a single dose of praziquantel in Nigerian schoolchildren. *PLoS Negl Trop Dis* 2: e241.

180. Barda B, Coulibaly JT, Puchkov M, Huwyler J, Hattendorf J, Keiser J, 2016. Efficacy and safety of moxidectin, synriam, synriam-praziquantel versus praziquantel against Schistosoma haematobium and S. mansoni infections: a randomized, exploratory phase 2 trial. *PLoS Negl Trop Dis* 10: e0005008.

181. Sissoko MS et al., 2009. Efficacy of artesunate sulfamethoxypyrazine/pyrimethamine versus praziquantel in the treatment of Schistosoma haematobium in children. *PLoS One* 4.

182. Soukhathammavong PA, Sayasone S, Phongluxa K, Xayaseng V, Utzinger J, Vounatsou P, Hatz C, Akkhavong K, Keiser J, Odermatt P, 2012. Low efficacy of single-dose albendazole and mebendazole against hookworm and effect on concomitant helminth infection in Lao PDR. *PLoS Negl Trop Dis* 6: e1417.

183. Albonico M, Mathema P, Montresor A, Khakurel B, Reggi V, Pandey S, Savioli L, 2007. Comparative study of the quality and efficacy of originator and generic albendazole for mass treatment of soil-transmitted nematode infections in Nepal. *Trans R Soc Trop Med Hyg* 101: 454–460.

184. Steinmann P, Utzinger J, Du ZW, Jiang JY, Chen JX, Hattendorf J, Zhou H, Zhou XN, 2011. Efficacy of single-dose and triple-dose albendazole and mebendazole against soil-transmitted helminths and Taenia spp.: a randomized controlled trial. *PLoS One* 6.

185. Belizario VY, Amarillo ME, de Leon WU, de los Reyes AE, Bugayong MG, Macatangay BJ, 2003. A comparison of the efficacy of single doses of albendazole, ivermectin, and

diethylcarbamazine alone or in combinations against Ascaris and Trichuris spp. *Bull World Health Organ* 81: 35–42.

186. Ndyomugyenyi R, Kabatereine N, Olsen A, Magnussen P, 2008. Efficacy of ivermectin and albendazole alone and in combination for treatment of soil transmitted helminths in pregnancy and adverse events: a randomized open label controlled intervention trial in Masindi District, Western Uganda. *Am J Trop Med Hyg* 79: 856–863.

187. Adegnika AA et al., 2014. Randomized, controlled, assessor-blind clinical trial to assess the efficacy of single- versus repeated-dose albendazole to treat Ascaris lumbricoides, Trichuric trichiura, and hookworm infection. *Antimicrob Agents Chemother* 58: 2535–2540.

188. Legesse M, Erko B, Medhin G, 2004. Comparative efficacy of albendazole and three brands of mebendazole in the treatment of ascariasis and trichuriasis. *East Afr Med J* 81: 134–138.

189. Speich B, Ame SM, Ali SM, Alles R, Hattendorf J, Utzinger J, Albonico M, Keiser J, 2012. Efficacy and safety of nitazoxanide, albendazole, and nitazoxanide-albendazole against Trichuris trichiura infection: a randomized controlled trial. *PLoS Negl Trop Dis* 6: 1–8.

190. Albonico M, Bickle Q, Montresor A, Savioli L, Taylor M, 2003. Efficacy of mebendazole and levamisole alone or in combination against intestinal nematode infections after repeated targeted mebendazole treatment in Zanzibar. *Bull World Health Organ* 81: 343–352.

191. Ekenjoku AJ, Oringangi C, Meremikwu MM, 2013. Comparative efficacy of levamisole, mebendalzole and pyrantel pamoate against common intestinal nematodes among children in Calabar, South-South Nigeria. *Niger J Paed* 40: 217–221.

192. Adams VJ, Lombard CJ, Dhansay MA, Markus MB, Fincham JE, 2004. Efficacy of albendazole against the whipworm trichuris trichiura - a randomised, controlled trial. *S Afr Med J* 94: 972–976.

193. Steinmann P, Zhou XN, Du ZW, Jiang JY, Xiao SH, Wu ZX, Zhou H, Utzinger J, 2008. Tribendimidine and albendazole for treating soil-transmitted helminths, Strongyloides stercoralis and Taenia spp.: open-label randomized trial. *PLoS Negl Trop Dis* 2: 1-10.

194. Mekonnen Z, Levecke B, Boulet G, Bogers JP, Vercruysse J, 2013. Efficacy of different albendazole and mebendazole regimens against heavy-intensity Trichuris trichiura infections in school children, Jimma Town, Ethiopia. *Pathog Glob Health* 107: 207–209.

195. Bartoloni A, Guglielmetti P, Cancrini G, Gamboa H, Roselli M, Nicoletti A, Paradisi F, 1993. Comparative efficacy of a single 400 mg dose of albendazole or mebendazole in the treatment of nematode infections in children. *Trop Geogr* 45: 114–116.

196. Jongsuksuntigul P, Jeradit C, Pornpattanakul S, Charansri U, 1993. A comparative study on the efficacy of albendazole and mebendazole in the treatment of ascariasis hookworm infection and trichuriasis. *Southeast Asian J Trop Med Public Health* 24: 724–729.

197. Muchiri EM, Thiong'o FW, Magnussen P, Ouma JH, 2001. A comparative study of different albendazole and mebendazole regimens for the treatment of intestinal infections in school children of Usigu division, Western Kenya. *J Parasitol* 87: 413–418.

198. Wen LY, Yan XL, Sun FH, Fang YY, Yang MJ, Lou LJ, 2008. A randomized, double-blind, multicenter clinical trial on the efficacy of ivermectin against intestinal nematode infections in China. *Acta Trop* 106: 190–194.

199. Horton J, 2000. Albendazole: a review of anthelmintic efficacy and safety in humans. *Parasitology* 121: S113–132.

200. Speich B et al., 2015. Efficacy and safety of albendazole plus ivermectin, albendazole plus mebendazole, albendazole plus oxantel pamoate, and mebendazole alone against Trichuris trichiura and concomitant soil-transmitted helminth infections: a four-arm, randomised controlled trial. *Lancet Infect Dis* 15: 277–284.

201. Knopp S et al,, 2010. Albendazole and mebendazole administered alone or in combination with ivermectin against Trichuris trichiura: a randomized controlled trial. *Clin Infect Dis* 51: 1420–1428.

202. Silber SA et al., 2017. Efficacy and safety of a single-dose mebendazole 500 mg chewable, rapidly-disintegrating tablet for Ascaris lumbricoides and Trichuris trichiura infection treatment

in pediatric patients: a double-blind, randomized, placebo-controlled, phase 3 study. *Am J Trop Med Hyg* 97: 1851–1856.

203. Speich B, Moser W, Ali SM, Ame SM, Albonico M, Hattendorf J, Keiser J, 2016. Efficacy and reinfection with soil-transmitted helminths 18-weeks post-treatment with albendazole-ivermectin, albendazole-mebendazole, albendazole-oxantel pamoate and mebendazole. *Parasit Vectors* 9: 123.

204. Barda B, Ame SM, Ali SM, Albonico M, Puchkov M, Huwyler J, Hattendorf J, Keiser J, 2018. Efficacy and tolerability of moxidectin alone and in co-administration with albendazole and tribendimidine versus albendazole plus oxantel pamoate against Trichuris trichiura infections: a randomised, non-inferiority, single-blind trial. *Lancet Infect Dis* 18: 864–873.

205. Anto EJ, Nugraha SE, 2019. Efficacy of albendazole and mebendazole with or without levamisole for Ascariasis and Trichuriasis. *Open Access Maced J Med Sci* 7: 1299–1302.

206. Moser W et al., 2017. Efficacy and safety of tribendimidine, tribendimidine plus ivermectin, tribendimidine plus oxantel pamoate, and albendazole plus oxantel pamoate against hookworm and concomitant soil-transmitted helminth infections in Tanzania and Côte d'Ivoire: a randomised, controlled, single-blinded, non-inferiority trial. *Lancet Infect Dis* 17: 1162–1171.

207. Moser W, Ali SM, Ame SM, Speich B, Puchkov M, Huwyler J, Albonico M, Hattendorf J, Keiser J, 2016. Efficacy and safety of oxantel pamoate in school-aged children infected with Trichuris trichiura on Pemba Island, Tanzania: a parallel, randomised, controlled, dose-ranging study. *Lancet Infect Dis* 16: 53–60.

208. Wimmersberger D, Coulibaly JT, Schulz JD, Puchkow M, Huwyler J, N'Gbesso Y, Hattendorf J, Keiser J, 2018. Efficacy and safety of ivermectin against Trichuris trichiura in preschool-aged and school-aged children: a randomized controlled dose-finding trial. *Clin Infect Dis* 67: 1247–1255.

209. Palmeirim MS, Ame SM, Ali SM, Hattendorf J, Keiser J, 2018. Efficacy and safety of a single dose versus a multiple dose regimen of mebendazole against hookworm Infections in children: a randomised, double-blind trial. *EClinicalMedicine* 1: 7–13.

210. Keller L, Palmeirim MS, Ame SM, Ali SM, Puchkov M, Huwyler J, Hattendorf J, Keiser J, 2020. Efficacy and safety of ascending dosages of moxidectin and moxidectin-albendazole against Trichuris trichiura in adolescents: a randomized controlled trial. *Clin Infect Dis* 70: 1193–1201.

211. Patel C, Coulibaly JT, Schulz JD, N'Gbesso Y, Hattendorf J, Keiser J, 2020. Efficacy and safety of ascending dosages of albendazole against *Trichuris trichiura* in preschool-aged children, school-aged children and adults: a multi-cohort randomized controlled trial. *EClinicalMedicine* 22: 100335.

212. Husin N, Pasaribu AP, Ali M, Suteno E, Wijaya W, Pasaribu S, 2020. Comparative efficacy and reinfection of albendazole-mebendazole, albendazole-pyrantel pamoate, and mebendazole on soil-transmitted helminths. *Maced J Med Sci* 8: 978–982.

213. Sapulete EJJ, de Dwi Lingga Utama IMG, Sanjaya Putra IGN, Kanya Wati D, Arimbawa IM, Gustawan IW, 2020. Efficacy of albendazole-pyrantel pamoate compared to albendazole alone for *Trichuris trichiura* infection in children: a double blind randomised controlled trial. *Malays J Med Sci* 27: 67–74.

214. Sungkar S et al., 2019. The effectiveness of triple-dose albendazole in comparison with mebendazole for the treatment of trichuriasis in children. *Int J Appl Pharm* 11: 104–107.

215. Olsen A, Namwanje H, Nejsum P, Roepstorff A, Thamsborg SM, 2009. Albendazole and mebendazole have low efficacy against Trichuris trichiura in school-age children in Kabale District, Uganda. *Trans R Soc Trop Med Hyg* 103: 443–446.

216. Diawara A et al., 2013. Association between response to albendazole treatment and β-tubulin genotype frequencies in soil-transmitted helminths. *PLoS Negl Trop Dis* 7: e2247.

217. Samuel F, Degarege A, Erko B, 2014. Efficacy and side effects of albendazole currently in use against Ascaris, Trichuris and hookworm among school children in Wondo Genet, Southern Ethiopia. *Parasitol Int* 63: 450–455.

218. Lubis IN, Pasaribu S, Lubis CP, 2012. Current status of the efficacy and effectiveness of albendazole and mebendazole for the treatment of Ascaris lumbricoides in North-Western Indonesia. *Asian Pac J Trop Med* 5: 605–609.

219. Adugna S, Kebede Y, Moges F, Tiruneh M, 2007. Efficacy of mebendazole and albendazole for Ascaris lumbricoides and hookworm infections in an area with long time exposure for antihelminthes, Northwest Ethiopia. *Ethiop Med J* 45: 301–306.

220. Raccurt CP, Lambert MT, Bouloumie J, Ripert C, 1990. Evaluation of the treatment of intestinal helminthiases with albendazole in Djohong (North Cameroon). *Trop Med Parasitol* 41: 46–48.

221. Gyorkos TW, Maheu-Giroux M, Blouin B, Saavedra L, Casapía M, 2013. Efficacy of a single dose of albendazole for soil-transmitted helminth infections in school children of a village in Iquitos, Perú. *Rev Peru Med Exp Salud Publica* 30: 601–607

222. Mihrshahia S, Caseya GJ, Montresorb A, Phucc TQ, Thachc DTC, Tienc NT, Biggsa BA, 2009. The effectiveness of 4 monthly albendazole treatment in the reduction of soil-transmitted helminth infections in women of reproductive age in Vietnam. *Int J Parasitol* 39: 1037–1043.

223. van der Werff SD, Vereecken K, van der Laan K, Campos Ponce M, Junco Díaz R, Núñez FA, Rivero LR, Gorbea MB, Polman K, 2014. Impact of periodic selective mebendazole treatment on soil-transmitted helminth infections in Cuban schoolchildren. *Trop Med Int Health* 19: 706–718.

224. Albonico M, Smith PG, Hall A, Chwaya HM, Alawi KS, Savioli L, 1994. A randomized controlled trial comparing mebendazole and albendazole against Ascaris, Trichuris and hookworm infections. *Trans R Soc Trop Med Hyg* 88: 585–589.

225. Chan L, Kan SP, Bundy DA, 1992. The effect of repeated chemotherapy on age-related predisposition to Ascaris lumbricoides and Trichuris trichiura. *Parasitology* 104: 371–377.

226. Hall A, Nahar Q, 1994. Albendazole and infections with Ascaris lumbricoides and Trichuris trichiura in children in Bangladesh. *Trans R Soc Trop Med Hyg* 88: 110–112.

227. Oyewole F, et al., 2007. Helminthic reduction with albendazole among school children in riverine communities of Nigeria. *J Rural Health* 6: 6–10.

228. Upatham ES, Viyanant V, Brockelman WY, Kurathong S, Lee P, Chindaphol U, 1989. Prevalence, incidence, intensity and associated morbidity of intestinal helminths in south Thailand. *Int J Parasitol* 19: 217–228.

229. Wang BR, Wang HC, Li LW, Zhang XL, Yue JQ, Wang GX, Shi XQ, Xiao FR, 1987. Comparative efficacy of thienpydin, pyrantel pamoate, mebendazole and albendazole in treating ascariasis and enterobiasis. *Chin Med J (Engl)* 100: 928–930.

230. Rafi S, Memon A, Billo AG, 1997. Efficacy and safety of mebendazole in children with worm infestation. *J Pak Med Assoc* 47: 140–141.

231. Kirwan P, Asaolu SO, Molloy SF, Abiona TC, Jackson AL, Holland CV, 2009. Patterns of soil-transmitted helminth infection and impact of four-monthly albendazole treatments in preschool children from semi-urban communities in Nigeria: a double-blind placebo-controlled randomised trial. *BMC Infect Dis* 9: 20.

232. Kumar H, Jain K, Jain R, 2014. A study of prevalence of intestinal worm infestation and efficacy of anthelminthic drugs. *Med J Armed Forces India* 70: 144–148.

233. Seo BS, Cho SY, Chai JY, 1978. Reduced Single Dose Of Mebendazole In Treatment Of Ascaris Lumbricoides Infection. *Kisaengchunghak Chapchi* 16: 21–25.

234. Abadi K, 1985. Single dose mebendazole therapy for soil-transmitted nematodes. *Am J Trop Med Hyg* 34: 129–33.

235. Ash A, Okello A, Khamlome B, Inthavong P, Allen J, Thompson RCA, 2017. Controlling Taenia solium and soil transmitted helminths in a northern Lao PDR village: Impact of a triple dose albendazole regime. *Acta Trop* 174: 171–178.

236. Mekonnen Z, Meka S, Ayana M, Bogers J, Vercruysse J, Levecke B, 2013. Comparison of individual and pooled stool samples for the assessment of soil-transmitted helminth infection intensity and drug efficacy. *PLoS Negl Trop Dis* 7: e2189.

237. Kale OO, 1977. A comparative trial of the anthelminthic efficacy of pyrantel pamoate (Combantrin) and thiabendazole (Mintezol). *Afr J Med Med Sci* 6: 89–93.

238. Chege SW, Gitoho F, Wanene GS, Mwega VJ, Rees PH, Kinyanjui H, 1974. Single dose treatment of hookworm in Murang'a district. *East Afr Med J* 51: 60–62.

239. Farid Z, Bassily S, Miner WF, Hassan A, Laughlin LW, 1977. Comparative single/dose treatment of hookworm and roundworm infections with levamisole, pyrantel and bephenium*. *J Trop Med Hyg* 80: 107–108.

240. Kurup R, Hunjan GS, 2010. Epidemiology and control of Schistosomiasis and other intestinal parasitic infections among school children in three rural villages of south Saint Lucia. *J Vector Borne Dis* 47: 228–234.

241. Vercruysse J, et al, 2011. Assessment of the anthelmintic efficacy of albendazole in school children in seven countries where soil-transmitted helminths are endemic. *PLoS Negl Trop Dis* 5: e948.

242. Norhayati M, Oothuman P, Azizi O, Fatmah MS, 1997. Efficacy of single dose albendazole on the prevalence and intensity of infection of soil-transmitted helminths in Orang Asli children in Malaysia. *Southeast Asian J Trop Med Public Health* 28: 563–569.

243. Ahmad A, Zohra A, Yasmin N, 1986. Albendazole in intestinal helminthiasis. *J Pak Med Assoc* 36: 114–117.

244. Maisonneuve H, Zribi M, Peyron F, 1984. A pediatric suspension of albendazole in the treatment of ascariasis ancylostomiasis and trichuriasis (167 Patients). *Curr Ther Res Clin Exp* 36: 404–408.

245. Gazder AJ, Roy J, 1987. Albendazole suspension in the treatment of intestinal helminthiasis in children. *Curr Ther Res Clin Exp* 41: 324–327.

246. Misra PK, Pande NK, Jagota SC, 1985. Albendazole in the treatment of intestinal helminthiasis in children. *Curr Med Res Opin* 9: 516–519.

247. Rahman WA, 1996. Comparative trials using albendazole and mebendazole in the treatment of soil-transmitted helminths in schoolchildren on Penang, Malaysia. *Southeast Asian J Trop Med Public Health* 27: 765–767.

248. Maisonneuve H, Rossignol JF, Addo A, Mojon M, 1985. Ovicidal effects of albendazole in human ascariasis, ancylostomiasis and trichuriasis. *Ann Trop Med Parasitol* 79: 79–82.

249. Jagota SC, 1986. Albendazole, a broad-spectrum anthelmintic, in the treatment of intestinal nematode and cestode infection: a multicenter study in 480 patients. *Clin Ther* 8: 226–231.

250. Saif, M, 1984. Clinical trial in Egypt of albendazole as an intestinal anthelmintic agent. *J R Soc Med* 61: 37–43.

251. Zawde, D, 1987. The treatment of intestinal helminthiasis with albendazole. *Ethiop Med J* 25: 83–86.

252. Yongyuth P, Koyadun S, Jaturabundit N, Sampuch A, Bhumiratana A, 2006. Efficacy of a single-dose treatment with 300 mg diethylcarbamazine and a combination of 400 mg albendazole in reduction of Wuchereria bancrofti antigenemia and concomitant geohelminths in Myanmar migrants in Southern Thailand. *J Med Assoc Thai* 89: 1237–1248.

253. Siriaut C, Bhumiratana A, Koyadun S, Anurat K, Satitvipawee P, 2005. Short-term effects of treatment with 300 mg oral-dose diethylcarbamazine on nocturnally periodic Wuchereria bancrofti microfilaremia and antigenemia. *Southeast Asian J Trop Med Public Health* 36: 832–840.

254. Bhumiratana A, Pechgit P, Koyadun S, Siriaut C, Yongyuth P, 2010. Imported bancroftian filariasis: diethylcarbamazine response and benzimidazole susceptibility of Wuchereria bancrofti in dynamic cross-border migrant population targeted by the national program to eliminate Lymphatic Filariasis in South Thailand. *Acta Trop* 113: 121–128.

255. Yongyuth P, Koyadun S, Jaturabundit N, Jariyahuttakij W, 2007, Bhumiratana A. Adverse reactions of 300 MG diethylcarbamazine, and in a combination of 400 MG albendazole, for a mass annual single dose treatment, in migrant workers in Phang Nga province. *J Med Assoc Thai* 90: 552–563.

256. Koyadun S, Bhumiratana A, Prikchu P, 2003. Wuchereria bancrofti antigenemia clearance among Myanmar migrants after biannual mass treatments with diethylcarbamazine, 300 mg oral-dose FILADEC tablet, in Southern Thailand. *Southeast Asian J Trop Med Public Health* 34: 758–767.

257. Husin N, Pasaribu AP, Ali M, Suteno E, Wijaya W, Pasaribu S, 2020. Comparative efficacy and reinfection of albendazole-mebendazole, albendazole-pyrantel pamoate, and mebendazole on soil-transmitted helminths. *Maced J Med Sci* 8: 978–982.

258. Gultom DE, Ali M, Pasaribu AP, Pasaribu S, 2020. Two or three consecutive days albendazole treatment has better efficacy than single-dose albendazole treatment for trichuriasis. *Indones Biomed J* 12: 45–50.

259. Moser W, Sayasone S, Xayavong S, Bounheuang B, Puchkov M, Huwyler J, Hattendorf J, Keiser J, 2018. Efficacy and tolerability of triple drug therapy with albendazole, pyrantel pamoate, and oxantel pamoate compared with albendazole plus oxantel pamoate, pyrantel pamoate plus oxantel pamoate, and mebendazole plus pyrantel pamoate and oxantel pamoate against hookworm infections in school-aged children in Laos: a randomised, single-blind trial. *Lancet Infect Dis* 18: 729–737.

260. Speich B, Ame SM, Ali SM, Alles R, Huwyler J, Hattendorf J, Utzinger J, Albonico M, Keiser J, 2014. Oxantel pamoate-albendazole for Trichuris trichiura infection. *N Engl J Med* 370: 610–620.

261. Barda B, et al., 2017. Efficacy of Moxidectin Versus Ivermectin Against Strongyloides stercoralis Infections: A Randomized, Controlled Noninferiority Trial. *Clin Infect Dis* 65: 276–281.

262. Belew S, et al., 2015. Assessment of Efficacy and Quality of Two Albendazole Brands Commonly Used against Soil-Transmitted Helminth Infections in School Children in Jimma Town, Ethiopia. *PLoS Negl Trop Dis* 9: e0004057.

263. Tefera E, Belay T, Mekonnen SK, Zeynudin A, Belachew T, 2015. Therapeutic efficacy of different brands of albendazole against soil transmitted helminths among students of Mendera Elementary School, Jimma, Southwest Ethiopia. *Pan Afr Med J* 22: 252.

264. Albonico M, Bickle Q, Haji HJ, Ramsan M, Khatib KJ, Montresor A, Salvioli L, Taylor M, 2002. Evaluation of the efficacy of pyrantel-oxantel for the treatment of soil-transmitted nematode infections. *Trans R Soc Trop Med Hyg* 96: 685–690.

265. Marti H, Haji HJ, Savioli L, Chwaya HM, Mgeni AF, Ameir JS, Hatz C, 1996. A comparative trial of a single-dose ivermectin versus three days of albendazole for treatment of Strongyloides stercoralis and other soil-transmitted helminth infections in children. *Am J Trop Med Hyg* 55: 477–481.

266. Zeleke AJ, Bayih AG, Afework S, Gilleard JS, 2020. Treatment efficacy and re-infection rates of soil-transmitted helminths following mebendazole treatment in schoolchildren, Northwest Ethiopia. *Trop Med Health* 48: 90.

267. Kar SK, Dwibedi B, Kerketa AS, Maharana A, Panda SS, Mohanty PC, Horton J, Ramachandran CP, 2015. A randomized controlled trial of increased dose and frequency of albendazole with standard dose DEC for treatment of Wuchereria bancrofti microfilaremics in Odisha, India. *PLoS Negl Trop Dis* 9: e0003583.

268. Oqueka T, Supali T, Ismid IS, Purnomo, Rückert P, Bradley M, Fischer P, 2005. Impact of two rounds of mass drug administration using diethylcarbamazine combined with albendazole on the prevalence of Brugia timori and of intestinal helminths on Alor Island, Indonesia. *Filaria J* 13: 5.

269. Kilpatrick ME, Trabolsi B, Farid Z, 1981. Levamisole compared to mebendazole in the treatment of Ancylostoma duodenale in Egypt. *Trans R Soc Trop Med Hyg* 75: 578–579.

270. Miller MJ, Farahmandian I, Arfaa F, Katz N, Winsor E, Bennett E, 1978. An evaluation of levamisole for treatment of ascariasis. *South Med J* 71: 137–140

271. Xu LL, Jiang B, Duan JH, Zhuang SF, Liu YC, Zhu SQ, Zhang LP, Zhang HB, Xiao SH, Zhou XN, 2014. Efficacy and safety of praziquantel, tribendimidine and mebendazole in patients with co-infection of Clonorchis sinensis and other helminths. *PLoS Negl Trop Dis* 8: e3046.

272. Sur D, Saha DR, Manna B, Rajendran K, Bhattacharya SK, 2005. Periodic deworming with albendazole and its impact on growth status and diarrhoeal incidence among children in an urban slum of India. *Trans R Soc Trop Med Hyg* 99: 261–267.

273. Datry A, et al., 1994. Treatment of strongyloides stercoralis infection with ivermectin compared with albendazole: results of an open study of 60 cases. *Trans R Soc Trop Med Hyg* 88: 344-345.

274. Moulia-Pelat JP, Glaziou P, Weil GJ, Nguyen LN, Gaxotte P, Nicolas L, 1995. Combination ivermectin plus diethylcarbamazine, a new effective tool for control of lymphatic filariasis. *Trop Med Parasitol* 46: 9–12.

275. Ottesen EA, 1985. Efficacy of diethylcarbamazine in eradicating infection with lymphatic-dwelling filariae in humans. *Rev Infect Dis* 7: 341-356.

276. Kimura E, et. al., 1992. Long-term efficacy of single-dose mass treatment with diethylcarbamazine citrate against diurnally subperiodic Wuchereria bancrofti: eight years' experience in Samoa. *Bull World Health Organ* 70: 769–776.

277. Kimura E, Penaia L, Spears GF, 1985. The efficacy of annual single-dose treatment with diethylcarbamazine citrate against diurnally subperiodic bancroftian filariasis in Samoa. *Bull World Health Organ* 63: 1097–1106.

278. Koroma MM, Williams RA, de la Haye RR, Hodges M, 1996. Effects of albendazole on growth of primary school children and the prevalence and intensity of soil-transmitted helminths in Sierra Leone. *J Trop Pediatr* 42: 371–372.

279. Sirivichayakul C, Pojjaroen-anant C, Wisetsing P, Chanthavanich P, Praevanit R, Limkittikul K, Pengsaa K, 2001. A comparative trial of albendazole alone versus combination of albendazole and praziquantel for treatment of Trichuris trichiura infection. *Southeast Asian J Trop Med Public Health* 32: 297–301.

280. Mpairwe H, et al., 2011. Anthelminthic treatment during pregnancy is associated with increased risk of infantile eczema: randomised-controlled trial results. *Pediatr Allergy Immunol* 22: 305–312.

281. Scherrer AU, Sjöberg MK, Allangba A, Traoré M, Lohourignon LK, Tschannen AB, N'Goran EK, Utzinger J, 2009. Sequential analysis of helminth egg output in human stool samples following albendazole and praziquantel administration. *Acta Trop* 109: 226–231.

282. Webb EL, et al., 2011. Effect of single-dose anthelmintic treatment during pregnancy on an infant's response to immunisation and on susceptibility to infectious diseases in infancy: a randomised, double-blind, placebo-controlled trial. *Lancet* 377: 52–62.

283. Jamshidi M, Mohraz M, Zangeneh M, Jamshidi A, 2008. The effect of combination therapy with albendazole and praziquantel on hydatid cyst treatment. *Parasitol Res* 103: 195–199.

284. Nahmias J, Greenberg Z, Djerrasi L, Giladi L, 1991. Mass treatment of intestinal parasites among Ethiopian immigrants. *Isr J Med Sci* 27: 278–283.

285. Adenusi AA, Oke AO, Adenusi AO, 2003. Comparison of ivermectin and thiabendazole in the treatment of uncomplicated human Strongyloides stercoralis infection. *Afr J Biotechnol* 2: 465–469.

286. Gann PH, Neva FA, Gam AA, 1994. A randomized trial of single- and two-dose ivermectin versus thiabendazole for treatment of strongyloidiasis. *J Infect Dis* 169: 1076-1079.

287. Igual-Adell R, Oltra-Alcaraz C, Soler-Company E, Sánchez-Sánchez P, Matogo-Oyana J, Rodríguez-Calabuig D, 2004. Efficacy and safety of ivermectin and thiabendazole in the treatment of strongyloidiasis. *Expert Opin Pharmacother* 5: 2615–2619.

288. Bockarie MJ, Tavul L, Ibam I, Kastens W, Hazlett F, Tisch DJ, Alpers MP, Kazura JW, 2007. Efficacy of single-dose diethylcarbamazine compared with diethylcarbamazine combined with albendazole against Wuchereria bancrofti infection in Papua New Guinea. *Am J Trop Med Hyg* 76: 62–66.

289. Prasad R, Mathur PP, Taneja VK, Jagota SC, 1985. Albendazole in the treatment of intestinalhelminthiasis in children. *Clin Ther* 7: 164–168.

290. Flohr C, et al., 2007. Low efficacy of mebendazole against hookworm in Vietnam: two randomized controlled trials. *Am J Trop Med Hyg* 76: 732–736.

291. Gunawardena NK, Kumarendran B, Manaperi N, Senarathna BP, Silva M, Pathmeswaran A, de Silva, NR, 2013. Randomized placebo-controlled trial of the efficacy of mebendazole polymorphs in the treatment of hookworm infections. *Am J Trop Med Hyg* 11: 12.

292. Lim JK, 1975. Pyrantel Embonate and bephenium hydroxynaphthoate in the treatment of hookworm infection. *Kisaengchunghak Chapchi* 13: 19–30.

293. Morgan P, Yamamoto M, Teesdale CH, Pugh RN, 1983. Albendazole: a new treatment for hookworm. *Med Q J Med Assoc Malawi* 16: 4–5.

294. Pugh RN, Teesdale CH, Burnham GM, 1986. Albendazole in children with hookworm infection. *Ann Trop Med Parasitol* 80: 565-567.

295. Rossignol JF, Maisonneuve H, 1983. Albendazole: placebo-controlled study in 870 patients with intestinal helminthiasis. *Trans R Soc Trop Med Hyg* 77: 707–711.

296. Sacko M, De Clercq D, Behnke M, Gilbert FS, Dorny P, Vercruysse J, 1983. Comparison of the efficacy of mebendazole, albendazole, and pyrantel in treatment of human hookworm infections in the Southern Region of Mali, West Africa. *Trans R Soc Trop Med Hyg* 93: 195–203.

297. Albonico M, Smith PG, Ercole E, Hall A, Chwaya HM, Alawi KS, Savioli L, 1995. Rate of reinfection with intestinal nematodes after treatment of children with mebendazole or albendazole in a highly endemic area. *Trans R Soc Trop Med Hyg* 89: 538–541.

298. Sorensen E, 1996. The efficacy of three anthelmintic drugs given in a single dose. *Ceylon Med J* 41: 42–45.

299. Viravan C, Migasena S, Bunnag D, Harinasuta T, 1982. Clinical trial of albendazole in hookworm infection. *Southeast Asian J Trop Med Public Health* 13: 654–657

300. Clercq D, Sacko M, Behnke J, Gilbert F, Dorny P, Vercruysse J, 1997. Failure of mebendazole In treatment of human hookworm infections in the southern region of mali. *Am J Trop Med Hyg* 57: 25-30.

301. Keiser J, Utzinger J, 2008. Efficacy of current drugs against soil-transmitted helminth infections. *JAMA* 299: 1937–1947.

302. Olds GR, et al., 1999. Double-blind placebo-controlled study of concurrent administration of Albendazoleand praziquantel in schoolchildren with schistosomiasis and geohelminths. *J Infect Dis* 179: 996-1003.

303. Musgrave IA, Hawes RB, Jameson JL, Sloane RA, Quayle PA, 1979. Evaluation of a new antihelminthic for triichuriasis, hookworm, and stronglyloidiasis. *Med J Aust* 1: 403–405.

304. Charoenlarp P, Waikagul J, Muennoo C, Srinophakun S, Kitayapom D, 1993. Efficacy of single-dose mebendazole, polmorphic forms A and C, in the treatment of hookworm and Trichuris infections. *Southeast Asian J Trop Med Public Health* 24: 712–716.

305. Migasena S, Suntharasamai P. Harinasuta T, 1978. Mebendazole, tetrachlorethylene and pyrantel pamoate in the treatment of hookworm infection. *Ann Trop Med Parasitol* 72: 199-200.

306. Nontasut P, Singhasivanon V, Prarinyanuparp V, Chiamratana B, Sanguankiat S, Dekumyoy P, Setasuban P, 1989. Effect of single-dose Albendazole and single-dose Mebendazoleon Necator americanus. *Southeast Asian J Trop Med Public Health* 20: 237–242.

307. Ovedoff DL, 1984. Summary of albendazoletrials in south-east asia. *Royal Society of Medicine International Congress and Symposium Series* 61: 103–113.

308. Phuvanandh D, Dulyapiree Y, Chatisiri J, Panrong A, Tanskul P, Phuvanandh M, 1994. Efficacy of common broad spectrum anthelmintics against hookworm, Ascaris and Trichuris in Hat Yai district, Songkhla Province. *Thailand. J Med Assoc Thai* 77: 357–362.

309. Sitthicharconchai P, Kulkumthorn M, Akarabovorn P, 1984. Clinical trial of a 400 mg dose of Albendazolein hookworm infection in Chulalongkorn Hospital. *Chulal Med J* 28: 909–913.

310. Bennett A, Guyatt H, 2000. Reducing intestinal nematode infection: efficacy of albendazole and mebendazole. *Parasitol Today* 16: 71–74.

311. Cabada MM, Lopez M, Arque E, Clinton White A, 2014. Prevalence of soil-transmitted helminths after mass albendazole administration in an indigenous community of the Manu jungle in Peru. *Pathog Glob Health* 108: 200–205.

312. Chachage M, et al., 2014. Helminth-associated systemic immune activation and HIV co-receptor expression: response to albendazole/praziquantel treatment. *PLoS Negl Trop Dis* 8: e2755.

313. Hürlimann E, et al., 2014. Effect of deworming on school-aged children's physical fitness, cognition and clinical parameters in a malaria-helminth co-endemic area of Côte d'Ivoire. *BMC Infect Dis* 14: 1.

314. Kinung'hi, SM, Magnussen P, Kishamawe C, Todd J, Vennervald BJ, 2015. The impact of anthelmintic treatment intervention on malaria infection and anaemia in school and preschool children in Magu district, Tanzania: an open label randomised intervention trial. *BMC Infect Dis* 15: 136.

315. Siza JE, et al., 2015. Prevalence of Schistosomes and Soil-Transmitted Helminths and Morbidity Associated with Schistosomiasis among Adult Population in Lake Victoria Basin, Tanzania. *Korean J Parasitol* 53: 525–533.

316. Sunish IP, Rajendran R, Munirathinam A, Kalimuthu M, Kumar VA, Nagaraj J, Tyagi BK, 2015. Impact on prevalence of intestinal helminth infection in school children administered with seven annual rounds of diethyl carbamazine (DEC) with albendazole. *Indian J Med Res* 141: 330–339.

317. Mani TR, Rajendran R, Sunish IP, Munirathinam A, Arunachalam N, Satyanarayana K, Dash AP, 2004. Effectiveness of two annual, single-dose mass drug administrations of diethylcarbamazine alone or in combination with albendazole on soil-transmitted helminthiasis in filariasis elimination programme. *Trop Med Int Health* 9: 1030–1035.

318. Njomo DW, Tomono N, Muhoho N, Mitsui Y, Josyline KC, Mwandawiro CS, 2010. The adverse effects of albendazole and praziquantel in mass drug administration by trained schoolteachers. *Afr J Health Sci* 17: 3–4

319. Kihara JH, Muhoho N, Mjomo D, Mwobbia IK, Josyline K, Mitsui Y, Awazawa T, Amano T, Mwandawiro C, 2007. Drug efficacy of praziquantel and albendazole in school children in Mwea Division, Central Province, Kenya. *Acta Trop* 102: 165–171.

320. Ramalingam S, Sinniah B, Krishnan U, 1983. Albendazole, an effective single dose, broad spectrum anthelmintic drug. *Am J Trop Med Hyg* 32: 984-949.

321. Wesche D, Barnish G, 1994. A comparative study of the effectiveness of mebendazole (Janssen) and generically equivalent mebendazole (Nordia) in intestinal helminthiasis in Papua New Guinean children. *P N G Med J* 37: 7–11.

322. Ai-Issa T, Jafar HT, Hassan I, 1985. A field study in the treatment of Intestinal Helminthes by the drug Zentel. *Bull Endem Dis* 26: 81–91.

323. Bassily S, El-Masry NA, Trabolsi B, Farid Z, 1984. Treatment of ancylostomiasis and ascariasis with Albendazole. *Ann Trop Med Parasitol* 78: 81–82.

324. Bwibo NO, Pamba HO, 1982. Double-blind comparative study of albendazole and placebo in the treatment of intestinal helminths. *RSM Internat Congr and Symp* 57: 49–59.

325. Chien FL, Foon K, Hassan K, 1989. Efficacy of albendazole against the three common soil-transmitted helminthiases. *Trop Biomed* 6: 133–136.

326. Ismail MM, Premaratne UN, Suraweera MG, 1991. Comparative efficacy of single dose anthelmintics in relation to intensity of geohelminth infections. *Ceylon Med J* 36: 162–167.

327. Okelo GBA, 1978. Open and placebo-controlled studies of albendazole in the treatment of intestinal helminthiasis. *J R Soc Med* 61: 57-62.

328. Sinniah B, Chew PI, Subramaniam K, 1970. A comparative trial of albendazole, mebendazole, pyrantel pamoate and oxantel pyrantel pamoateagainst soil-transmitted helminthiases in schoolchildren. *Trop Biomed* 7: 129–134.

329. Stephenson LS, Latham MC, Kinoti SN, Kurz KM, Brigham H, 1990. Improvements in physical fitness of Kenyan schoolboys infected with hookworm, Trichuris trichiura and Ascaris lumbricoides following a single dose of albendazole. *Trans R Soc Trop Med Hyg* 84: 277–282.

330. El-Masry NA, Trabolsi B, Bassily S, Farid Z, 1983. Albendazole in the treatment of Ancylostoma duodenale and Ascaris lumbricoides infections. *Trans R Soc Trop Med Hyg* 77: 160–161.

331. Reich MR, Govindaraj R, Dumbaugh K, Yang B, Brinkmann A, El-Saharty S, 1998. International strategies for tropical disease treatments: Experiences with praziquantel. https://apps.who.int/iris/bitstream/handle/10665/63824/WHO_DAP_CTD_98.5.pdf. Accessed April 1, 2022.

332. Campbell WC, Burg RW, Fisher MH, Dybas RA, 1984. The Discovery of Ivermectin and Other Avermectins. in Pesticide Synthesis Through Rational Approaches. Magee PS, Kohn GK, Menn JJ, eds. Pesticide Synthesis Through Rational Approaches. Washington, DC: American Chemical Society, 5-20.

333. Gyurik RJ, Theodorides VJ, 1975. Methyl 5-propylthio-2-benzimidazolecarbamate. US Patent.

334. Lohr S, 1989. SmithKline, Beecham to Merge. New York, NY: The New York Times. Available at: https://www.nytimes.com/1989/04/13/business/smithkline-beecham-to-merge.html. Accessed April 1, 2022.

335. Moore SD, Waldholz M, Raghavan A, 2000. Glaxo Wellcome Agrees to Acquire SmithKline Beecham for $75.7 Billion. Available at: https://www.wsj.com/articles/SB948069657140115932. Accessed April 1, 2022.

336. Brady M, Global Alliance to Eliminate Lymphatic Filariasis., 2014. Seventh meeting of the Global Alliance to Eliminate Lymphatic Filariasis: reaching the vision by scaling up, scaling down, and reaching out. Parasit Vectors 23: 46.

337. Chandler AD, 2009. Shaping the Industrial Century: The Remarkable Story of the Evolution of the Modern Chemical and Pharmaceutical Industries. Cambridge, MA: Harvard University Press.

338. Federal Trade Commission (FTC), 1997. FTC Settles Charges with American Cyanamid; Agency Alleged Company Had Fixed Prices and Restricted Competition. Available at: https://www.ftc.gov/news-events/press-releases/1997/01/ftc-settles-charges-american-cyanamid-agency-alleged-company-had. Accessed April 1, 2022.

339. Petersen M, 2002. American Home Is Changing Name to Wyeth. New York, NY: The New York Times. Available at: https://www.nytimes.com/2002/03/11/business/american-home-is-changing-name-to-wyeth.html. Accessed April 1, 2022.

340. Sorkin AR, Wilson D, 2009. Pfizer Agrees to Pay $68 Billion for Rival Drug Maker Wyeth. New York, NY: The New York Times. Available at: https://www.nytimes.com/2009/01/26/business/26drug.html. Accessed April 1, 2022.

341. Gelder JLH, Roevens LFC, Raeymaekers, AHM, 1972. Benzimidazole carbamates. US Patent.

342. William Andrew Publishing, 2006. Pharmaceutical Manufacturing Encyclopedia, 3rd Edition.

Supplementary tables

Table S1. WHO recommended anthelmintic drugs for use in preventive chemotherapy

Disease	ALB	MBD	DEC	IVM	PZQ
Ascariasis	X	X		X	
Hookworm	X	X			
Lymphatic filariasis	X		X	X	
Onchocerciasis				X	
Schistosomiasis					X
Trichuriasis	X	X		X	

Table S2. Drugs, doses, implementation thresholds and regimens in preventive chemotherapy

Disease	Drugs and dosages	Frequency of intervention
Lymphatic filariasis (where onchocerciasis is co-endemic)	IVM (according to height) plus ALB 400 mg	Once a year
Lymphatic filariasis (where onchocerciasis is not co-endemic)	DEC 6 mg (using age as criterion for dose) plus ALB 400 mg	Once a year
Onchocerciasis	IVM (according to height)	Once a year
Schistosomiasis	PZQ 40 mg	According to prevalence of infection
Soil-transmitted helminthiasis	ALB 400 mg or MBD 500mg	According to prevalence of infection

Table S3. Preventive chemotherapy courses of action

Intervention	Diseases targeted	Drugs	Frequency of implementation
MDA1	LF or<br>LF and ONCHO or<br>LF and STH or<br>LF and ONCHO and STH	Ivermectin and albendazole, administered together	Repeated at yearly intervals
MDA2	LF or<br>LF and STH	Diethylcarbamazine and albendazole, administered together	Repeated at yearly intervals
MDA3	ONCHO	Ivermectin	Repeated at yearly intervals
T3	SCHIST and STH	Praziquantel and albendazole or mebendazole, administered together	Repeated at yearly intervals
T2	SCHIST	Praziquantel	T2 should take place once a year in high-risk communities, once every 2 years in moderate risk communities, and twice during the period of primary schooling age in low-risk communities
T3	STH	Albendazole or mebendazole	Once or twice per year

Table S4. Recommended treatment strategy for STH in preventive chemotherapy

Category	Prevalence of any STH infection among school-aged children	Action to be taken
High-risk community	≥50%	Treat all school-aged children (enrolled and not enrolled) twice each year
Low-risk community	≥20% and <50%	Treat all school-aged children (enrolled and not enrolled) once each year

Table S5. Accreditation list

Drug	Abbreviation	Expired	Originator	Reference
Praziquantel	PZQ	Yes	Bayer	Bayer officially developed the drug and it named Biltricide.[331]
Ivermectin	IVM	Yes	Merck	W. C. Campbell, R. W. Burg, M. H. Fisher, and R. A. Dyba discovered Ivermectin as part of research conducted for Merck Labs.[332]
Albendazole	ALB	Yes	GlaxoSmithKline	SmithKline Corporation originally patented Albendazole in 1975.[333] SmithKline then merged with Beecham in 1989 to form SmithKline Beecham.[334] SmithKline Beecham then merged with Glaxo Wellcome to become GlaxoSmithKline.[335]
Diethylcarbamazine	DEC	Yes	Pfizer	Yellaragrada Subb Rao first discovered DEC at Lederle Labs in 1946.[336] Lederle Labs was owned at the time by American Cyanamid.[337] American became a subsidiary of American Home Products Corp. in 1995.[338] American Home Products eventually changed its name to Wyeth, and Wyeth was subsequently acquired by Pfizer.[339,340]
Mebendazole	MBD	Yes	Johnson & Johnson	Janssen Pharmaceuticals originally patented Mebendazole 1969.[341,342] Johnson and Johnson acquired Janssen Pharmaceuticals in 1961.

Supplementary figures

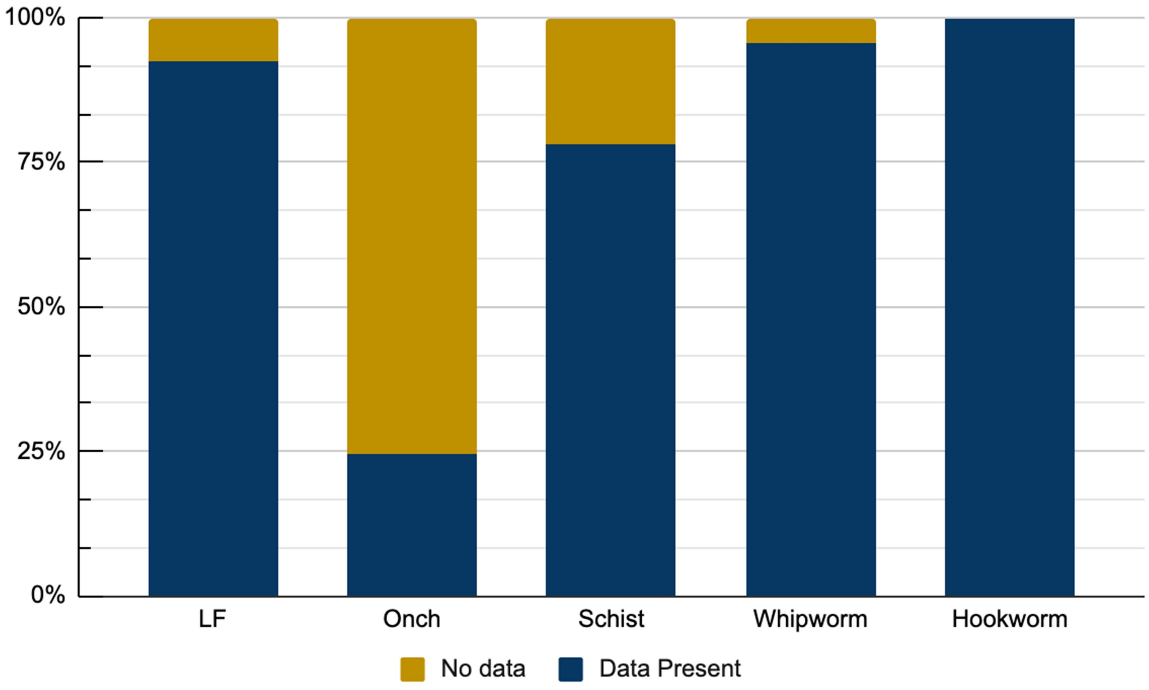

Figure S1. Daly data broken down by NTDs. Graph shows proportions of countries with DALY data by disease throughout 2015

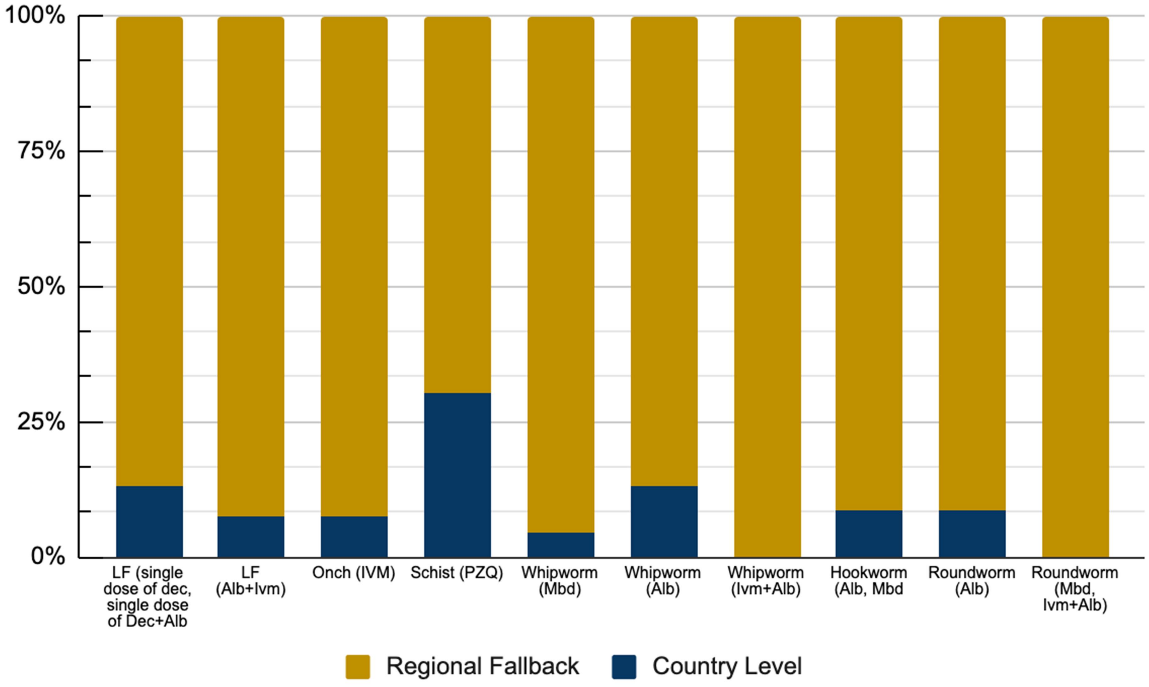

Figure S2. Efficacy data broken down by NTDs. Graph shows proportions of countries using country-level efficacy data vs. regional fallback data by regimen and disease throughout 2015

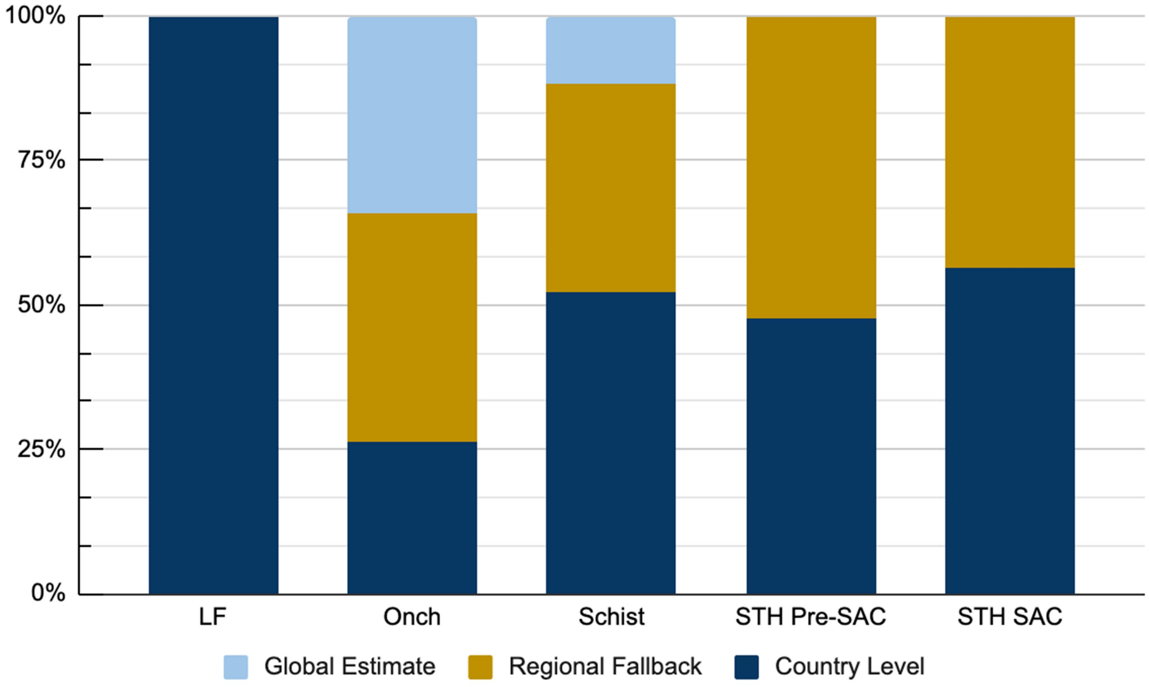

Figure S3. Treatment coverage percentage data availability. Data shows proportions of global estimate, regional fallback, and country level treatment coverage data present within diseases throughout 2015

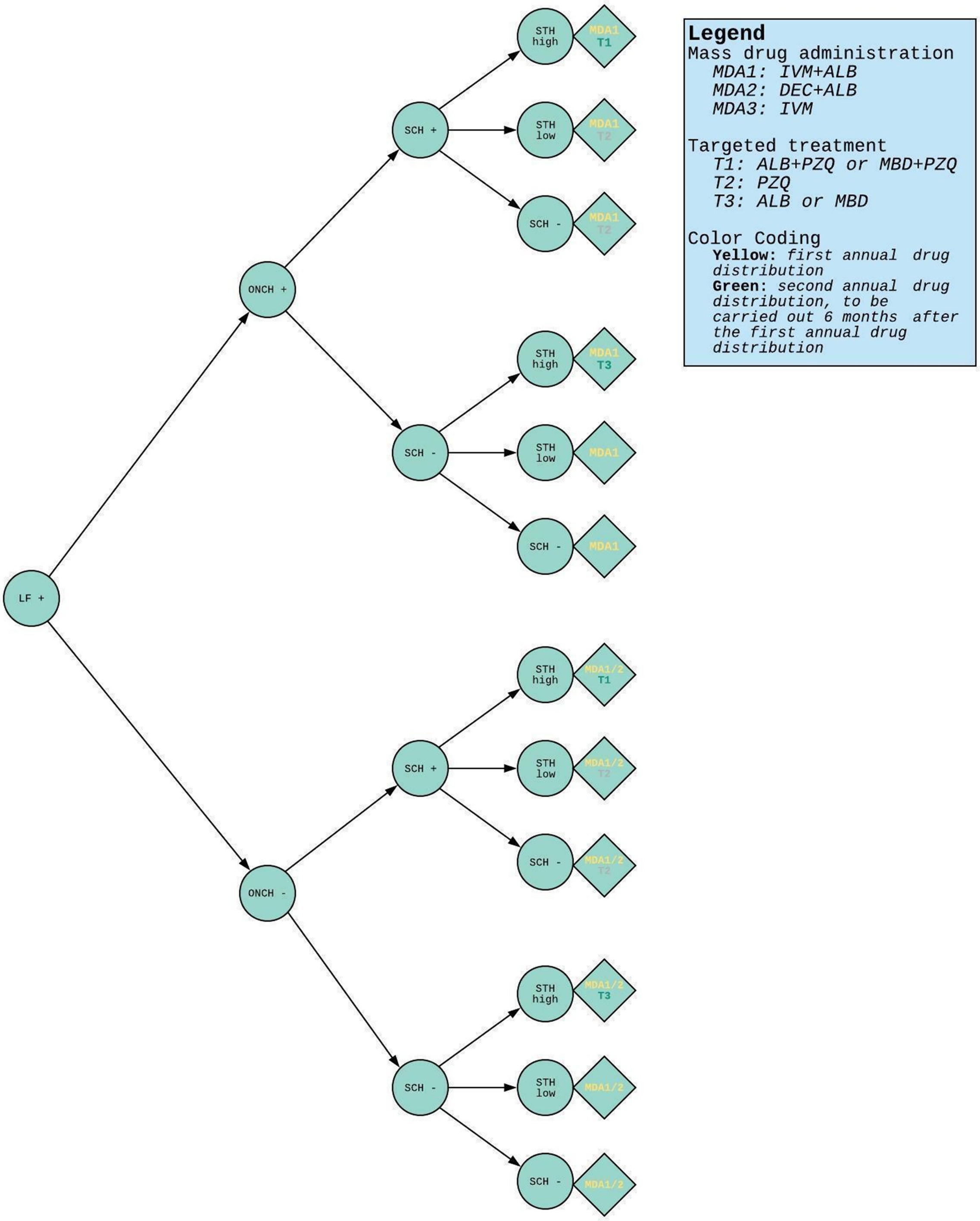

Figure S4. Intervention implementation in lymphatic filariasis endemic areas. Data mapping the breakdown comparisons in LF, Onch, and STH in regards to the first annual drug distribution vs. the second annual drug distribution.

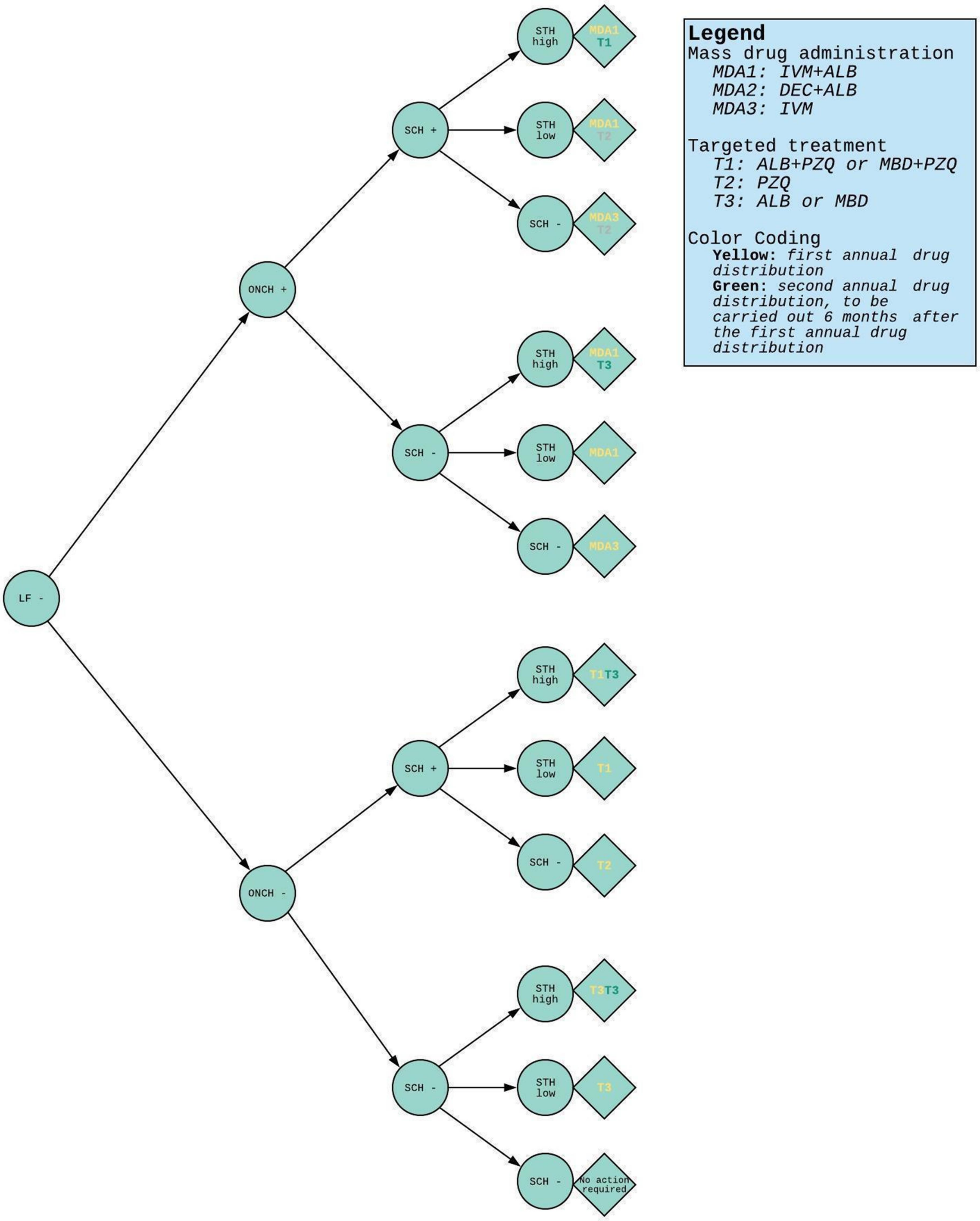

Figure S5. Intervention implementation in areas where lymphatic filariasis is not endemic. Data mapping the breakdown comparisons in LF, Onch, and STH where LF was not endemic in regards to the first annual drug distribution vs. the second annual drug distribution.

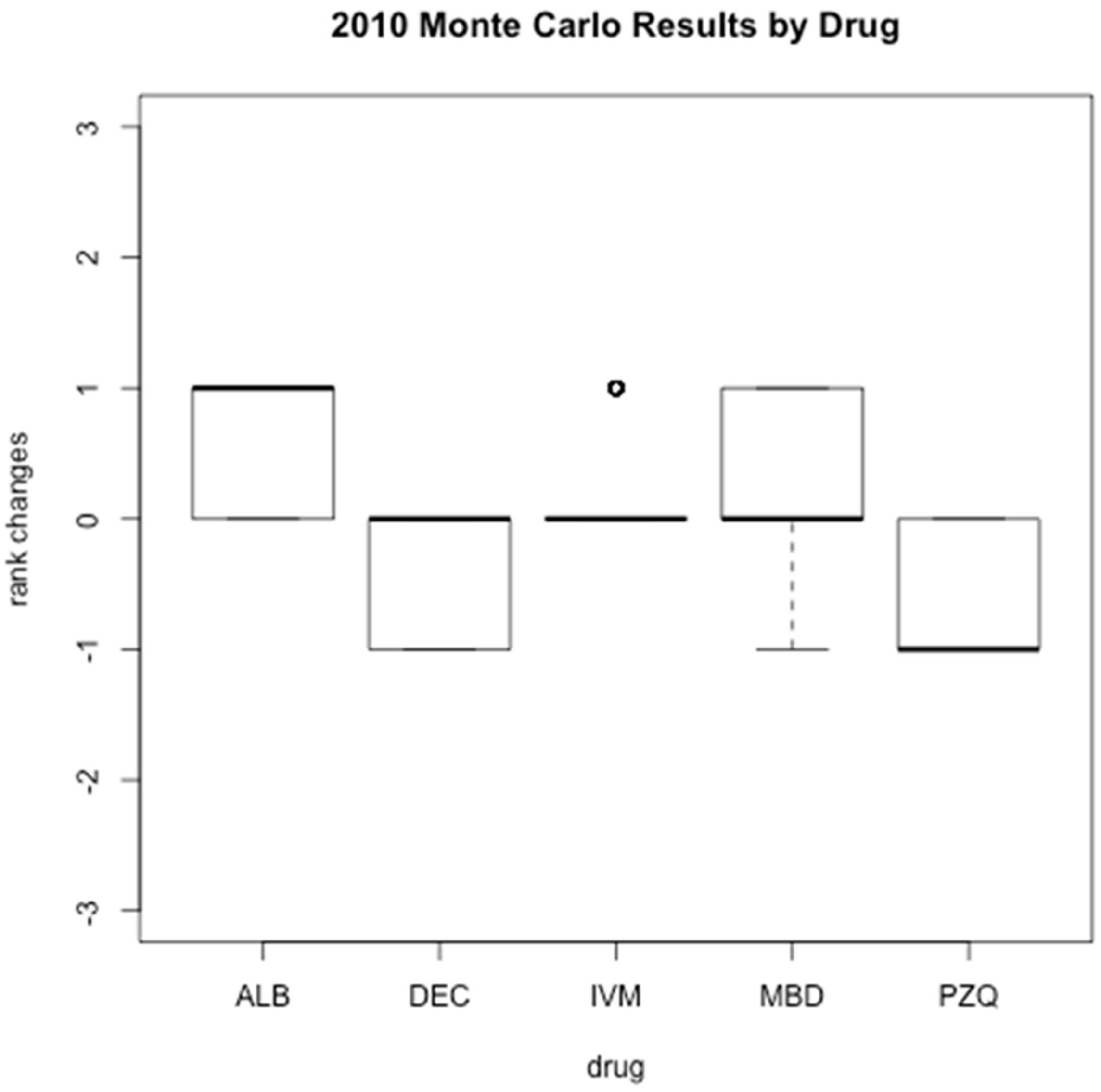

Figure S6. Monte Carlo results by drug, 2010. Changes in drug ranking in 2010. Note that albendazole's rank has a tendency to move up in 50% of cases and praziquantel's rank has a tendency to move down in 50% of cases. Similarly, mebendazole's rank has a tendency to move up in 50% of cases and diethylcarbamazine’s rank has a tendency to move down in 50% of cases and (though the former stayed within the interquartile range).

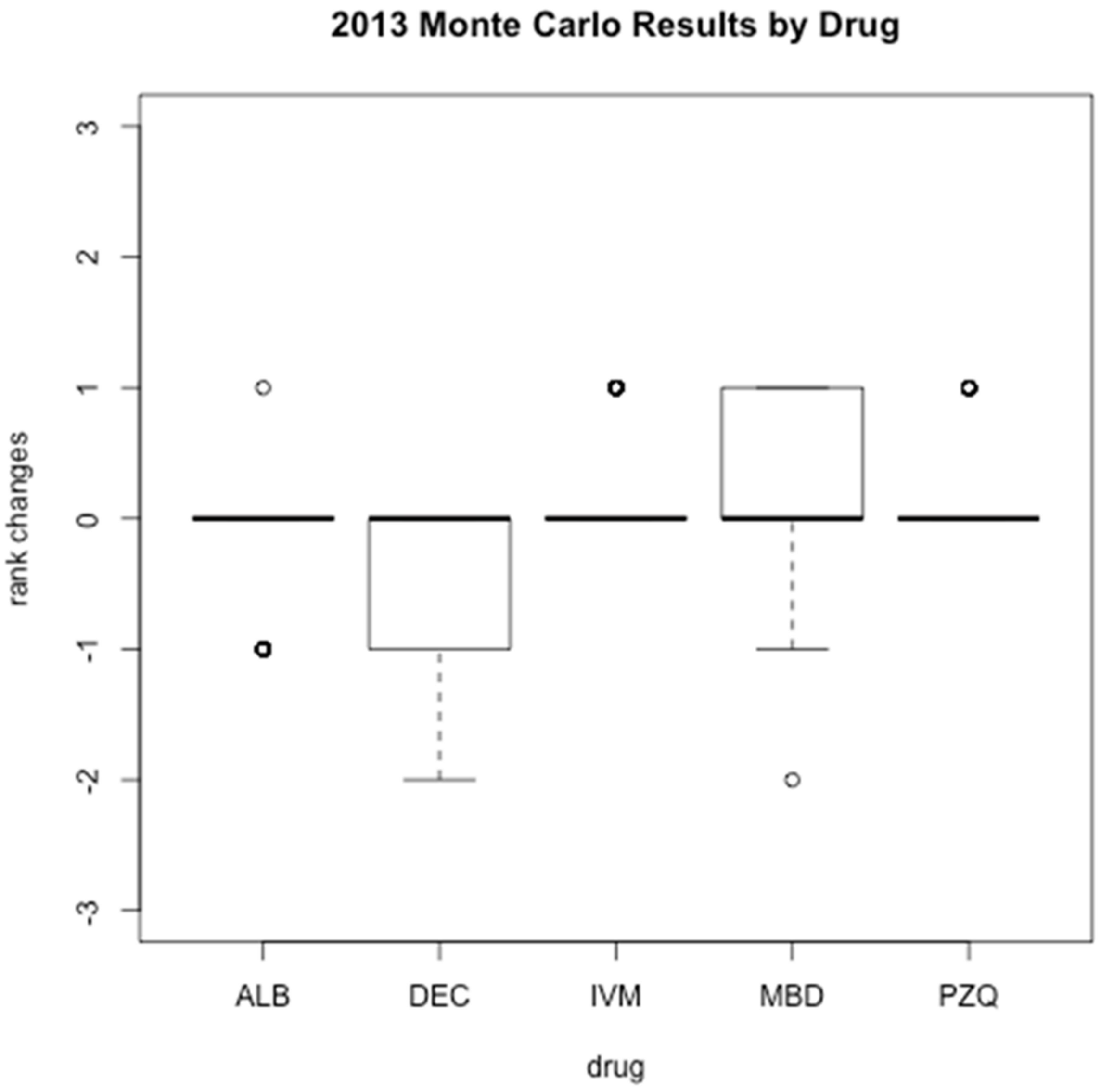

Figure S7. Monte Carlo results by drug, 2013. Changes in drug ranking in 2013. Note that mebendazole's rank has a tendency to move up in 50% of cases and diethylcarbamazine's rank has a tendency to move down in 50% of cases (though the later does exceed one positive interval change in the maximum case, it stayed within the interquartile range).

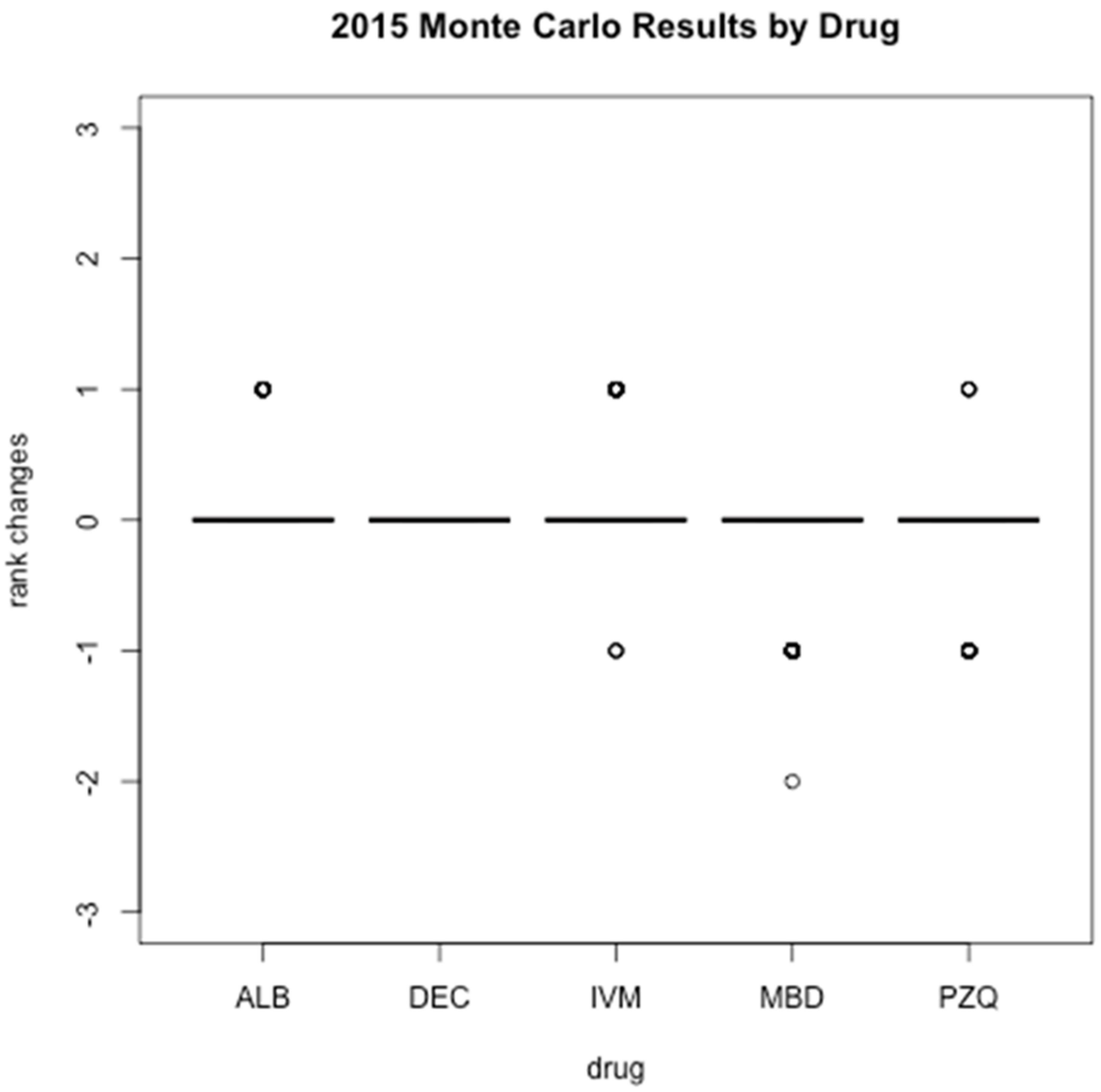

Figure S8. Monte Carlo results by drug, 2015. Changes in drug ranking in 2015. Note that all drugs held a stable ranking.

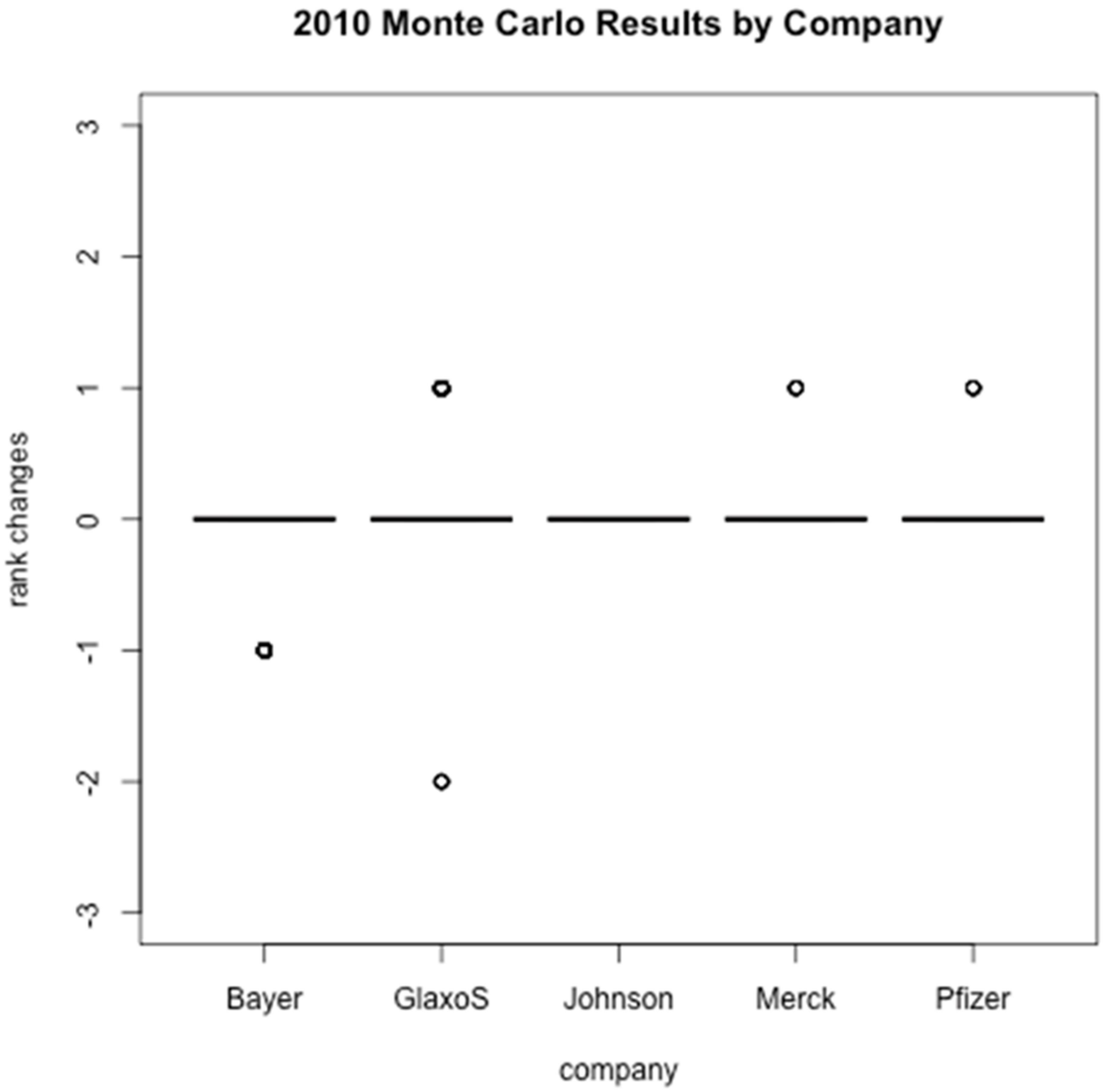

Figure S9. Monte Carlo results by company, 2010. Changes in company ranking in 2010. All companies held a stable ranking.

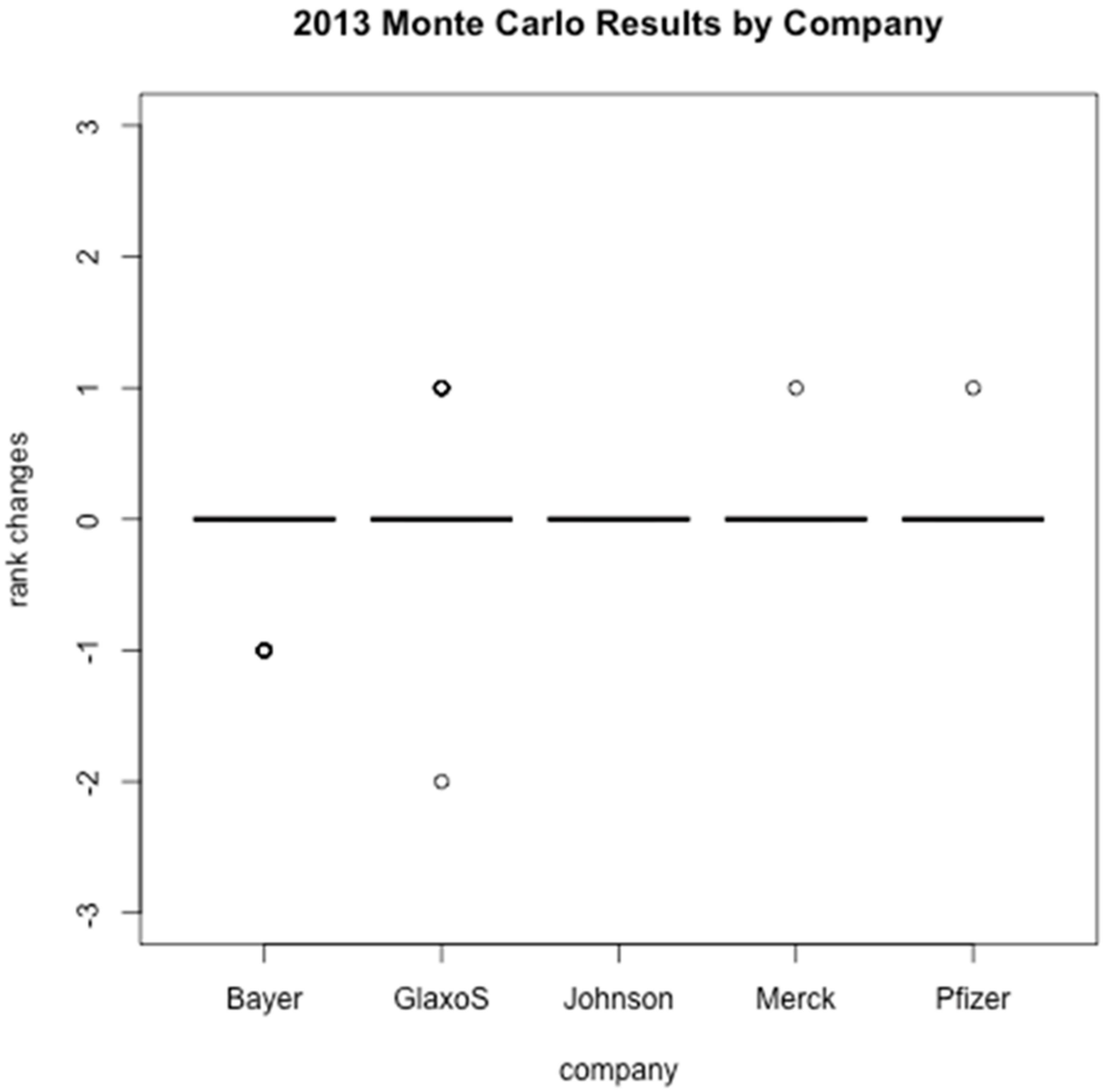

Figure S10. Monte Carlo results by company, 2013. Changes in company ranking in 2013. All companies held a stable ranking.

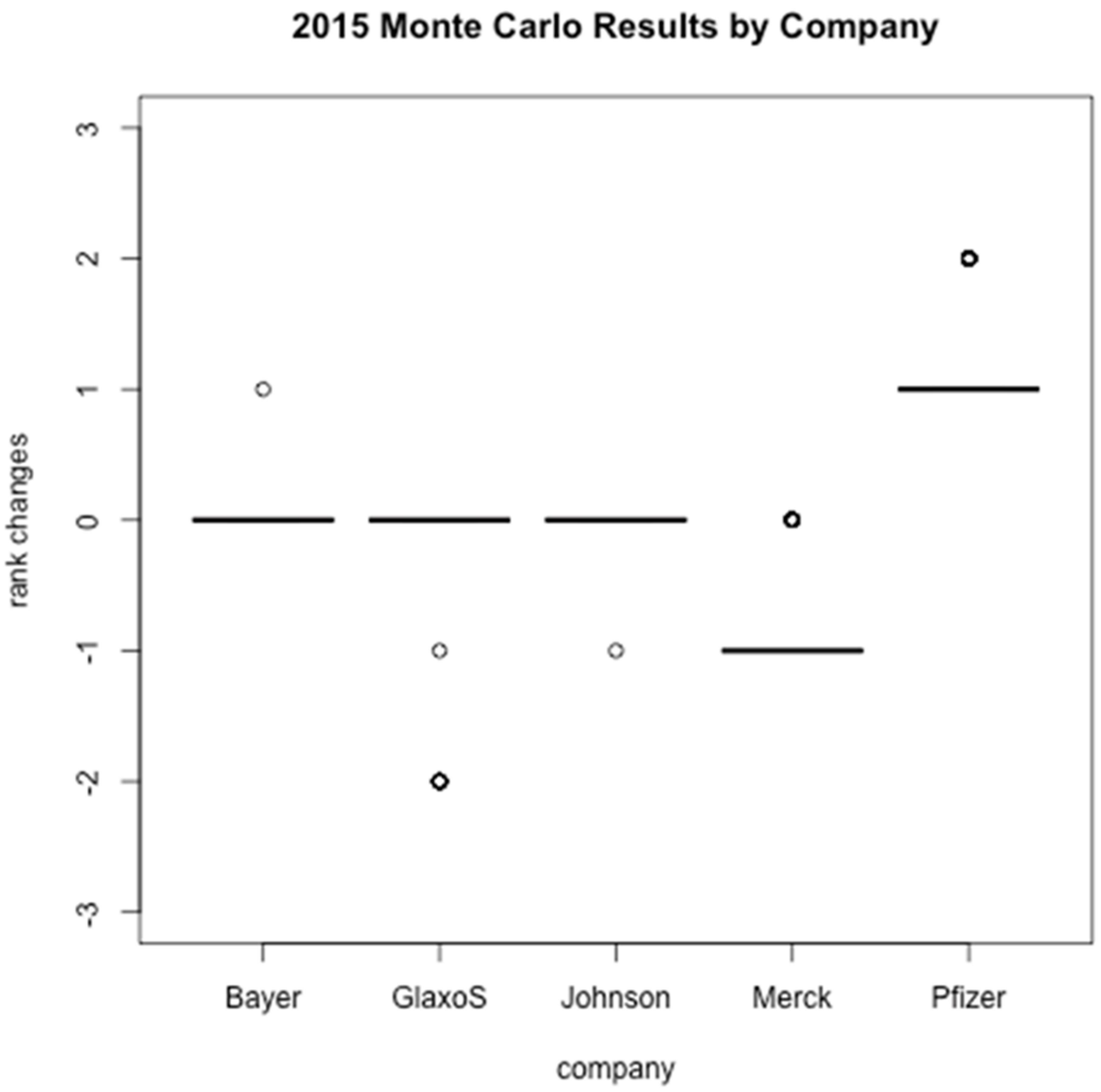

Figure S11. Monte Carlo results by company, 2015. Changes in company ranking in 2015. Merck went down one rank while Pfizer went up but overall the model was stable.

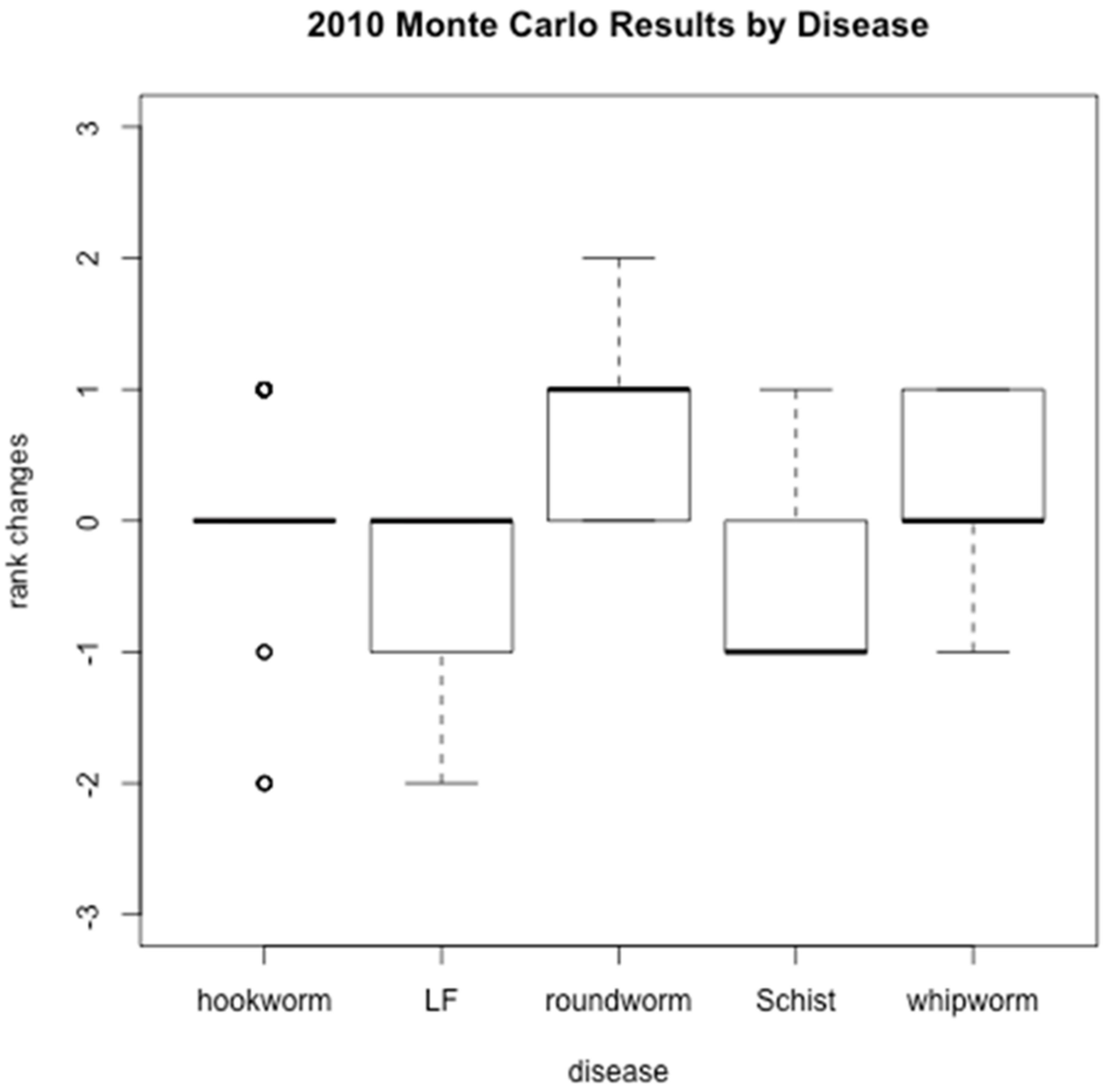

Figure S12. Monte Carlo results by disease, 2010. Changes in disease ranking in 2010. Note that roundworm and whipworm's rank tended to move up in 50% of cases and schistosomiasis' and lymphatic filariasis' rank tended to move down in 50% of cases. Although lymphatic filariasis' and roundworms' rank does exceed one positive interval change in the maximum case, the interquartile range remains within our acceptable bounds of stability.

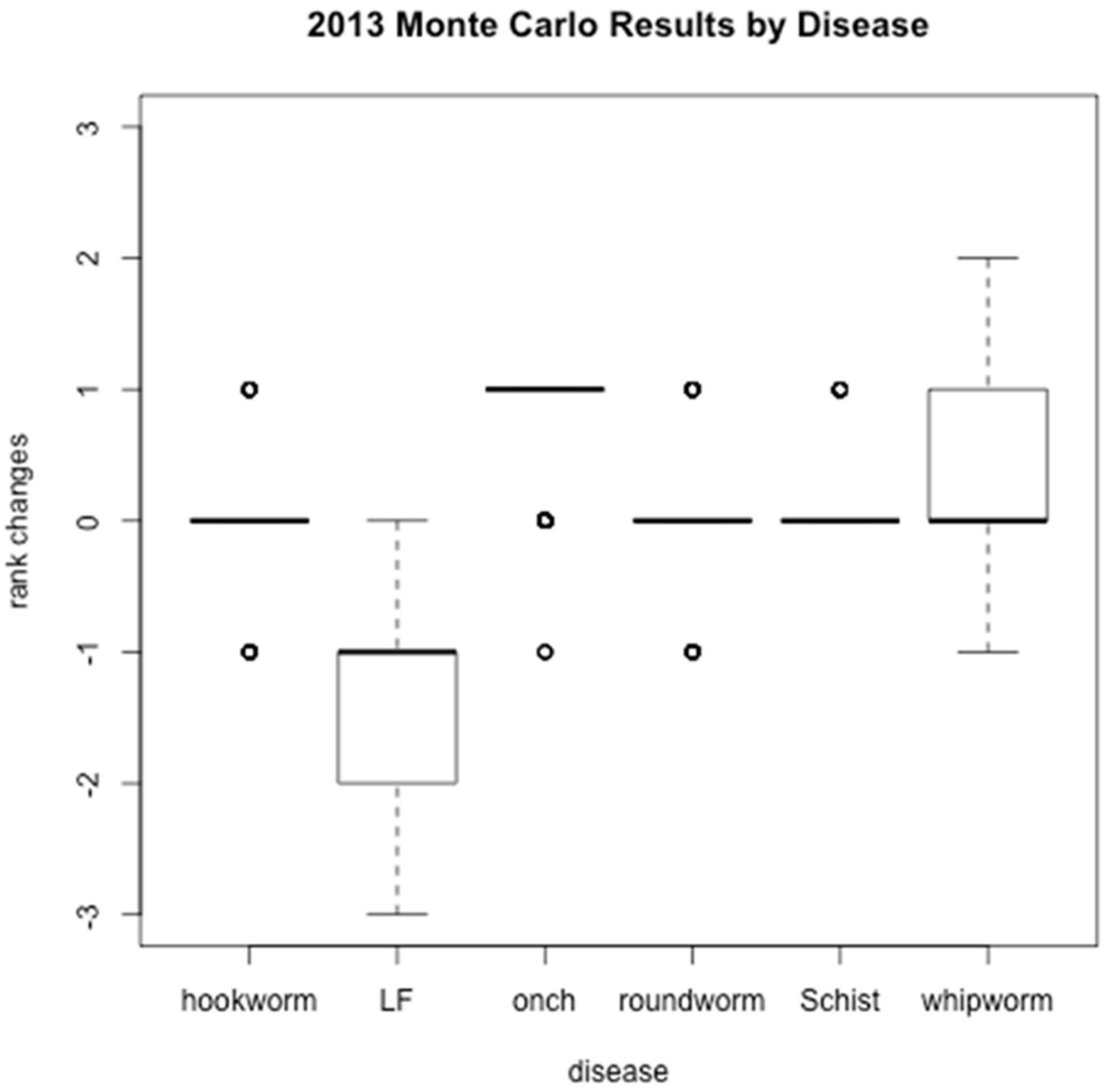

Figure S13. Monte Carlo results by disease, 2013. Changes in disease ranking in 2013. Note that onchocerciasis' rank tended to go up in 50% of cases and lymphatic filariasis' rank tended to go down in 50% of cases, although the later does exceed one positive interval change in the maximum case, both whipworm and lymphatic filariasis stayed in the interquartile range.

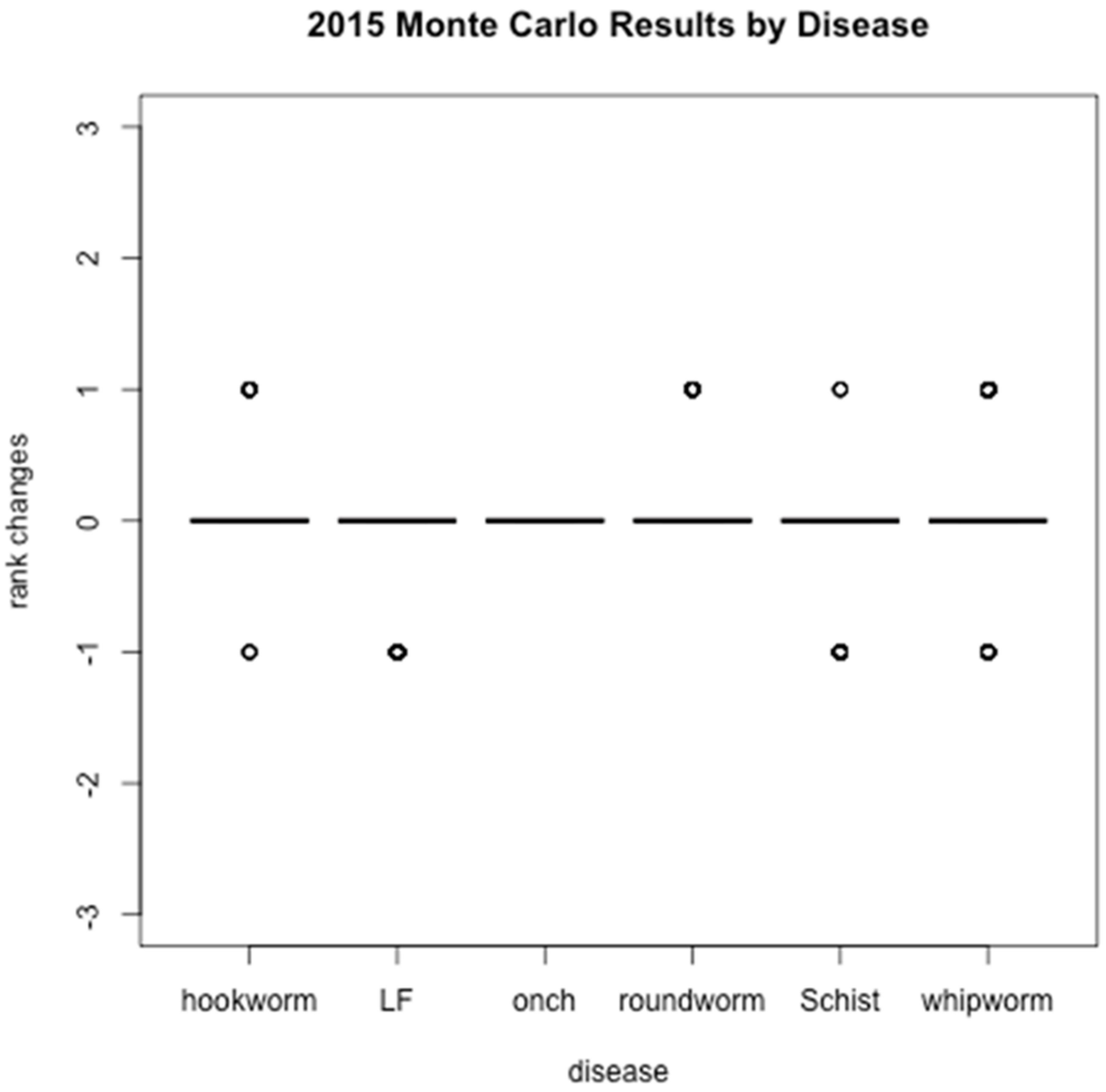

Figure S14. Monte Carlo results by disease, 2015. Changes in disease ranking in 2015. All diseases held a stable ranking.
